# Trust Beyond Accuracy: Conformal Uncertainty Quantification Reveals the Generalization Gap in Polymer Glass Transition Temperature Prediction

**DOI:** 10.1021/acsomega.6c02607

**Published:** 2026-07-23

**Authors:** Murat Akdoğan

**Affiliations:** Chemical Engineering Department, Hacettepe University, Ankara 06800, Turkey

## Abstract

Glass transition temperature (*T*_g_) is a key thermophysical property in polymer informatics, yet many machine learning (ML) studies focus on point prediction accuracy without explicitly evaluating reliability under chemical novelty. Here, we evaluate two established descriptor-based regressors, gradient-boosted trees (XGBoost) and support vector regression (SVR), on a 410-sample simulation-derived polymer data set using stratified, scaffold-based, and fingerprint-clustered validation regimes. We combine learning curves, applicability-domain diagnostics, split conformal prediction (SCP), subgroup coverage analysis, model-specific descriptor-importance analysis, and interval-aware triage metrics. Performance degraded and variability increased under novelty-enforcing splits, with SVR showing more stable point-prediction behavior than XGBoost in several regimes; this trend is interpreted as benchmark-specific, not as general model-class superiority. Conformal intervals maintained near-nominal marginal coverage but were often too wide for fine-grained candidate ranking, and subgroup diagnostics revealed local reliability limitations in low-similarity, high-*T*_g_, or chemistry-specific subsets. Thus, conformal intervals are best interpreted as conservative risk indicators for uncertainty-aware triage, not as high-resolution screening tools. Descriptor-importance analyses highlighted chemically plausible feature families related to topology, polarity, surface area, heteroatom content, and electronic-state descriptors, but these attributions are treated as model-level plausibility diagnostics, not physical validation of *T*_g_ mechanisms. Overall, this work provides a reproducible reliability-assessment workflow for small-data polymer *T*_g_ prediction against MD-derived labels, with experimental validation required before deployment against measured *T*_g_ data.

## Graphical abstract

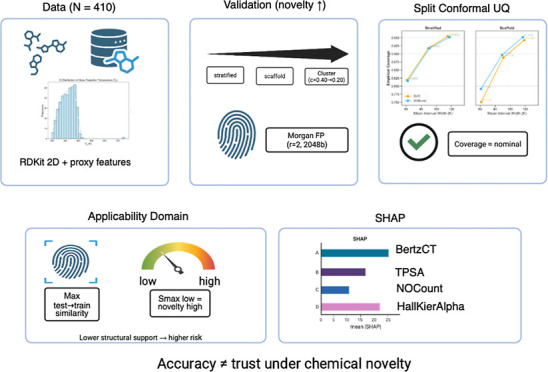

## 1 Introduction

Polymers are widely used across various industrial and technological applications, including electronics, medicine, energy storage and packaging due to their superior properties such as low cost, low density, resistance to corrosion, resistance to most chemicals and ease of manufacturing.^1^ However, the structural complexity and morphological diversity of polymers have posed significant obstacles to their development.^2^ The design and development of advanced polymeric materials with tailored properties remains one of the most challenging frontiers in materials science.^3,4^ Data-driven techniques and machine learning are increasingly being used in materials research to achieve significant time and cost savings compared to traditional experimental methods, which are often time-consuming and rely on trial-and-error approaches. These advanced methods accelerate the development and discovery of materials by learning structure–property relationships.^2,5–7^ With rapid advancement in machine learning (ML) and artificial intelligence (AI) applications, polymer informatics is an emerging discipline that enables rapid prediction of polymer structure–property relationships from a data science viewpoint.^8,9^ Polymer informatics has evolved from simple quantitative structure–property relationships (QSPR) models to complex architectures capable of navigating vast chemical spaces. Within this field, the glass transition temperature (*T*_g_) represents a critical thermal property, marking the transition of amorphous materials from a hard, brittle glassy state to a soft, leathery rubbery state.^10,11^ This thermodynamic parameter fundamentally governs polymer processability, mechanical performance, and end-use suitability across diverse applications ranging from heat-resistant aerospace composites to biocompatible drug-delivery matrices.^10,12–16^ Because *T*_g_ marks the onset of segmental chain mobility, it serves as a critical determinant of polymer performance, including strength and impact resistance, and defines the operational limits of the material’s service temperature range.^17^

The “generalization gap” remains a significant challenge in polymer informatics, primarily due to the historical reliance on random data set partitioning.^18^ By maintaining high structural similarity between training and evaluation sets, such strategies often yield overoptimistic performance metrics that frequently mask a model’s inability to generalize to novel chemical architectures during prospective discovery.^19,20^ Here, we use the term “generalization gap” operationally to denote the decrease in predictive performance and/or increase in predictive uncertainty observed when a model is evaluated under novelty-enforcing split regimes relative to an interpolative stratified split. Thus, the term does not refer only to a formal train-test loss gap, but to the practical degradation in model reliability when test polymers have reduced structural support from the training set.

In response, nonrandom validation regimes, particularly Bemis-Murcko scaffold^21^ splitting and fingerprint-based cluster splitting, are used here as operational stress tests for reduced train-test similarity and potential distribution shift, not as complete simulations of prospective polymer deployment. Scaffold splitting prohibits test-set molecular frameworks from appearing in training data, thereby enforcing structural dissimilarity at the level of core scaffolds and consistently revealing substantial degradation in predictive accuracy (*R* ^2^), error-ranking performance (Spearman ρ), and uncertainty calibration compared to random splits.^22^ This structural isolation effectively unmasks the overoptimistic performance estimates inherent in conventional random validation.^20^ However, scaffold splitting is not a complete solution for rigorous out-of-distribution (OOD) assessment. Due to the persistence of shared substructures and local chemical environments across distinct scaffolds, this regime often maintains a moderate, yet potentially misleading correlation between in-distribution and out-of-distribution performance.^19,23^ Consequently, models that appear robust on scaffold benchmarks may still exhibit substantial performance degradation when applied to chemical families bearing minimal structural precedent in the training set, motivating the use of even more stringent cluster-based evaluations. Cluster-based splitting enforces maximal structural dissimilarity by partitioning the data set via fingerprint-based distances, constructing test environments that approximate the extrapolative demands of prospective discovery.^24^ This approach places test compounds in chemical neighborhoods with limited precedent in training data, thereby testing model behavior under reduced structural support. Accordingly, cluster-based splits^24,25^ provide operational stress tests for reliability under reduced train-test similarity, not definitive measures of prospective extrapolation capability.

In materials discovery, reliance on point predictions alone is increasingly insufficient: false positives incur costly experimental waste, while false negatives discard promising candidates. Consequently, uncertainty quantification (UQ) has emerged as essential for distinguishing trustworthy predictions from high-risk extrapolations, transforming model outputs into actionable confidence estimates that enable rational prioritization of experimental resources.^26^ Several well-established UQ methods, such as Bayesian inference and ensemble-based resampling techniques, have been widely adopted for molecular property prediction.^27^ However, Bayesian methods impose substantial computational demands and can encounter identifiability issues that yield intractable multimodal posteriors,^28,29^ while ensemble-based resampling techniques lack formal coverage guarantees and can produce miscalibrated uncertainty estimates under distribution shift.^30^ To address these limitations, conformal prediction (CP) offers a theoretically grounded alternative by providing distribution-free UQ with guaranteed finite-sample coverage, regardless of the underlying model or data distribution.^31,32^ Specifically, SCP provides distribution-free prediction intervals that achieve user-specified marginal coverage under exchangeability, without parametric assumptions, making it well-suited to materials discovery where prediction reliability directly influences experimental decisions.^33,34^

In this study, we present a reproducible evaluation workflow for reliability-aware polymer *T*_g_ prediction in a small, simulation-derived data set. This study does not aim to establish a state-of-the-art polymer *T*_g_ predictor or to exhaustively compare all modern polymer ML architectures. Rather than introducing a new machine learning algorithm, UQ method, or polymer representation, we use two established small-data baseline models, SVR and XGBoost, to examine how validation regime, chemical novelty, applicability-domain (AD) diagnostics, and conformal uncertainty jointly affect reliability assessment. Specifically, we systematically combine (i) novelty-aware validation regimes based on stratified, scaffold-based, and fingerprint-clustered splits; (ii) AD diagnostics based on structural similarity and descriptor-space support; (iii) SCP for UQ; and (iv) model-specific descriptor-importance analyses, including XGBoost SHAP and SVR permutation importance, used as plausibility checks within the adopted repeat-unit feature space. The resulting workflow is designed as a reproducible evaluation protocol that can be extended to graph neural networks, directed message-passing architectures, polymer-specific representations, multimodal models, pretrained representations, and uncertainty-aware deep learning methods. The goal is not to construct a complete polymer-physics model of *T*_g_, but to provide a transparent evaluation benchmark for assessing reliability, uncertainty, and screening risk in data-sparse polymer informatics.

## 2 Materials and Methods

### 2.1 Data Set and Molecular Representation

We used a curated data set of 410 polymer repeat-unit structures characterized by molecular dynamics simulations.^35,36^ Each entry includes canonical SMILES, glass transition temperature (*T*_g_), cohesive energy density (*E*_coh_), radius of gyration (*R*_g_), and density (ρ). The target property, *T*_g_, exhibits a broad distribution ranging from 196.49 to 754.46 K (mean = 317.88 ± 54.95 K), covering both commodity and high-*T*_g_ polymers (Table 1).

**Table 1:** Summary Statistics of the Glass Transition Temperature (*T*_g_ *,* K) for the Curated Polymer Dataset (*N* = 410)^a^

# of data	mean	std	median	IQR	min	max
410	317.88	54.95	319.64	81.43	196.49	754.46

a Reported values include the mean, standard deviation, median, interquartile range (IQR), and the minimum–maximum range, highlighting the broad and heavy-tailed *T*_g_ distribution used for benchmarking model performance and uncertainty under increasing chemical novelty.

The present data set is derived from molecular dynamics (MD) simulations rather than experimental *T*_g_ measurements. Accordingly, this study evaluates model generalization and uncertainty calibration relative to a fixed MD-derived *T*_g_ target space, not experimental ground truth. We therefore do not interpret near-nominal conformal coverage against these labels as evidence of direct experimental validity. This distinction is important because simulated *T*_g_ can depend on force field parametrization, thermal protocol, and the fitting/analysis procedure used to extract *T*_g_ from the simulation trajectory.

Repeat-unit SMILES were featurized in RDKit (v2023.09.1)^37^ using two complementary input spaces: (i) standard RDKit 2D descriptors (Descriptors._desclist), capturing topological and physicochemical properties (e.g., logP, TPSA, heavy atom count);^38,39^ and (ii) a small set of polymer proxy features (e.g., ring counts, heteroatom fractions) designed to encode coarse structural signals that may be underrepresented by single-repeat descriptors. The custom proxy-feature list, definitions, and units are provided in Table S1. Missing descriptor values were imputed using medians computed only from the corresponding training partition within each cross-validation fold. This representation is based on single-repeat-unit chemistry and therefore does not explicitly encode molecular weight, degree of polymerization, dispersity, tacticity/stereoregularity, branching architecture, cross-link density, crystallinity, copolymer sequence, intermolecular packing, morphology, or processing history. These factors can influence *T*_g_ and may vary independently of repeat-unit chemistry. Consequently, the models developed here should be interpreted as learning correlations between repeat-unit descriptors, coarse proxy features, and MD-derived *T*_g_ labels rather than complete polymer-physics relationships.

Separately from predictive features, Morgan fingerprints (radius 2, 2048 bits)^40^ were computed only for (i) defining cluster-based splits and (ii) quantifying structural novelty via maximum test-to-train similarity (*S*_max_). This separation avoids conflating the predictive representation with the novelty diagnostics used to interpret generalization.

### 2.2 Data Splitting Strategies

To evaluate model behavior under validation regimes imposing different forms of train-test separation, we considered three splitting regimes and evaluated each using 5-fold cross-validation repeated over three random seeds (42/43/44) (15 test evaluations per regime).

### 2.3 Stratified Splitting

*T*_g_ values were binned into 10 quantiles, and stratified K-fold cross-validation was applied to maintain a balanced *T*_g_ distribution across folds. If quantile binning failed due to insufficient unique values, standard K-fold splitting was used.

### 2.4 Scaffold Splitting

Bemis-Murcko scaffolds^21^ were computed using RDKit’s *MurckoScaffold* utilities,^41^ and repeat units assigned to the same scaffold were kept within the same fold to reduce framework-level leakage between training and test partitions. We use this split as an intermediate diagnostic stress test. For ring-containing, aromatic, heteroatomic, or mixed aromatic–aliphatic repeat units, Murcko scaffolds can retain chemically meaningful core ring/linker architectures that are relevant to repeat-unit rigidity and substituent effects. However, for flexible, acyclic, or weakly scaffolded repeat units, the scaffold abstraction may be less informative for *T*_g_-relevant novelty. Accordingly, scaffold-based results are interpreted together with fingerprint-cluster splits and *S*_max_-based similarity diagnostics rather than as a standalone measure of polymer novelty or prospective deployment performance. Scaffold diversity, scaffold-size distribution, singleton-scaffold fraction, empty/generic-scaffold fraction, and representative examples from the largest scaffold groups are reported in Table S2 to document both the chemical interpretability and the limitations of scaffold-based partitioning in the present data set.

### 2.5 Cluster-Based Splitting

Morgan fingerprints were clustered using Tanimoto similarity cutoffs of *c* = 0.20, 0.30, and 0.40. These cutoffs were selected as operational sensitivity levels to generate different degrees of similarity-based cluster granularity, rather than as universal polymer-specific novelty thresholds. Lower cutoffs produce broader clusters, whereas higher cutoffs produce more localized clusters; however, the effective difficulty of each split depends on the resulting cluster-size distribution, fold composition, and residual train-test similarity after fold assignment. Therefore, each cluster-based split was characterized post hoc using the number of clusters, cluster-size distribution, singleton-cluster fraction, fold-size distribution, fold-level *T*_g_ summaries, selected chemistry-summary statistics, *S*_max_ distributions, and low-*S*_max_ tail fractions (Table S3; Figure S1).

### 2.6 Machine Learning Models and Hyperparameter Optimization

We benchmarked two complementary descriptor-based regressors: gradient-boosted decision trees implemented with XGBoost^42^ and support vector regression (SVR) with a radial basis function (RBF) kernel.^43^ SVR and XGBoost were selected as established, computationally tractable, small-data-compatible baselines rather than as exhaustive or state-of-the-art polymer-property predictors. SVR provides a regularized kernel-based model suitable for descriptor-based learning in small data sets, whereas XGBoost provides a strong nonlinear tree-ensemble baseline for tabular molecular descriptors. Their use enables repeated nested validation, conformal calibration, learning curve analysis, AD diagnostics, and descriptor-importance analysis at manageable computational cost. We emphasize that model-specific conclusions are restricted to these two baseline models and the adopted descriptor representation.

Hyperparameters were optimized using Optuna^44^ with the Tree-structured Parzen Estimator (TPE) sampler. For each outer fold, Optuna minimized inner 3-fold cross-validated RMSE over 25 trials using only the outer-training data. The final model was then refit on the full outer-training partition and evaluated once on the held-out outer test fold. SVR used a preprocessing pipeline consisting of StandardScaler followed by RBF kernel SVR, whereas XGBoost used the scikit-learn API. Hyperparameter search spaces are reported in Table S4 (Software: Optuna v3.3.0, scikit-learn v1.1.2, XGBoost v1.7.3).

### 2.7 Learning Curve Analysis

To quantify sample efficiency, models were trained on subsampled fractions of each outer training fold (0.2, 0.4, 0.6, 0.8, and 1.0, where 1.0 denotes the full outer training split). For each fold/seed/fraction, training indices were sampled without replacement using a deterministic pseudorandom generator keyed to the seed, fold, and fraction, with a minimum subset size of 10. When conformal prediction was applied, each training subset was further divided into a model-fit set and a calibration set, with 20% reserved for calibration and a minimum calibration size of 10 samples when permitted by the available subset size. At low training fractions, the calibration set can become small because it is drawn from the available training subset. Small calibration sets lead to discrete empirical quantiles and can increase variability in interval width and empirical coverage. Therefore, conformal results at low training fractions were interpreted as exploratory diagnostics rather than as stable estimates of interval efficiency. Outer test folds remained isolated throughout subsampling, tuning, calibration, and evaluation.

### 2.8 Uncertainty Quantification via Split Conformal Prediction

We used SCP to construct distribution-free prediction intervals, which provide finite-sample marginal coverage under calibration-test exchangeability.^45–47^ For calibration points, nonconformity scores were defined as absolute residuals

1 $$r_{i}=\left|y_{i}-{ŷ}_{i}\right|$$

for a target miscoverage level α, the conformal half-width ${\mathrm{q̂}}_{\alpha }$ was computed from the empirical quantile of calibration residuals using

2 k=⌈(ncal+1)(1−α)⌉

with k clipped to [1, ncal]. Prediction intervals for a test instance x were then defined as

3 $$\left[{ŷ}_{\left(x\right)}-{\mathrm{q̂}}_{a},{ŷ}_{\left(x\right)}+{\mathrm{q̂}}_{a}\right]$$

Interval quality was evaluated using empirical coverage and mean interval width. Empirical coverage was defined as the fraction of test points whose true values fell within the prediction interval.

The finite-sample guarantee of SCP is marginal: averaged over future samples drawn exchangeably from the same distribution, the prediction interval covers the true value with probability at least 1– α. This does not imply uniformly accurate coverage for every chemical subgroup, *T*_g_ range, or novelty level. Moreover, novelty-enforcing splits may induce calibration-test distribution shift, so coverage was evaluated empirically across validation regimes. Therefore, in addition to reporting marginal empirical coverage, subgroup coverage was analyzed as a diagnostic for potential local reliability failures.

### 2.9 Applicability Domain and Chemical Similarity Analysis

For each outer evaluation, we computed the maximum test-to-train Tanimoto similarity for each test polymer using Morgan fingerprints

4 $$S_{\max}\left(x_{test}\right)=\max_{x_{train}\in D_{train}}T\left(FP\left(x_{test}\right),FP\left(x_{train}\right)\right)$$

where T is the Tanimoto coefficient^48^ and FP(·) is the Morgan fingerprint representation. Similarities were computed with RDKit’s BulkTanimotoSimilarity, and the maximum value *S*_max_ was retained. Low *S*_max_ indicates reduced nearest-neighbor structural support from the training chemistry and was therefore used as a first-order structural-similarity diagnostic rather than as a standalone AD measure.

However, *S*_max_ alone does not define the standalone AD because it does not directly quantify descriptor-space density, local prediction-risk structure, or target-space support. Therefore, for each test polymer, we computed three categories of AD diagnostics. First, structural support was quantified using maximum test-to-train Morgan-Tanimoto similarity (*S*_max_), k-nearest-neighbor similarity, and Tanimoto-neighborhood density. *S*_max_ was defined as the maximum Morgan-Tanimoto similarity between each test polymer and all polymers in the corresponding training fold. k-nearest-neighbor similarity was computed as the arithmetic mean of the top five Morgan-Tanimoto similarities to the training fold (k_NN = 5); when fewer than five training samples were available, k_NN was reduced to the available number of training samples. Tanimoto-neighborhood density was defined as the fraction of training polymers with Morgan-Tanimoto similarity ≥0.30 to the test polymer. Thus, this density metric represents the local fraction of structurally similar training neighbors rather than a raw neighbor count. Second, descriptor-space support was evaluated using standardized descriptor nearest-neighbor distance and PCA-space distance. Third, target-space support was evaluated retrospectively by comparing each test *T*_g_ value with the target distribution represented in the corresponding training fold. Target-space diagnostics were used only for retrospective error analysis.

To quantify the fractions of predictions outside the empirical AD, we defined low-support flags using structural and descriptor-space diagnostics. *S*_max_ < 0.30 was used as a fixed low-similarity threshold. Thresholds for kNN similarity, Tanimoto-neighborhood density, descriptor nearest-neighbor distance, and PCA-space distance were defined from empirical diagnostic distributions to provide comparable inside/outside partitions. A composite structural outside-AD flag was assigned when at least one structural or descriptor-space AD threshold was violated. Retrospective target-space diagnostics, including target-space nearest-neighbor distance and outside-training *T*_g_ range indicators, were analyzed separately and were not used as prospective screening criteria. These thresholds are empirical diagnostics and should not be interpreted as universal polymer AD boundaries.

### 2.10 Subgroup Coverage Diagnostics

To assess local reliability beyond marginal coverage, empirical coverage was evaluated across predefined subgroups. Subgroups were defined using three criteria: (i) maximum test-to-train similarity, *S*_max_, divided into empirical low-, medium-, and high-similarity tertiles across all conformal-interval records; (ii) observed test *T*_g_ values, divided into empirical tertiles across all conformal-interval records; and (iii) broad repeat-unit chemistry descriptors, including ring-containing versus acyclic, aromatic versus nonaromatic, and heteroatom-rich versus heteroatom-poor repeat units, where subgroup size was sufficient for descriptive coverage estimation. Heteroatom-rich repeat units were defined as those with heteroatom fraction, calculated as noncarbon, non-hydrogen heavy atoms divided by total heavy atoms, at or above the data set median. For each subgroup, we report subgroup size, empirical coverage, target coverage, coverage gap, defined as empirical coverage minus target coverage, and mean interval width. These analyses are interpreted as diagnostics of local reliability.

### 2.11 Model-Specific Descriptor-Importance Analysis

To obtain model-specific descriptor-importance diagnostics, we used two complementary approaches. For XGBoost, Shapley Additive exPlanations (SHAP) were computed using TreeExplainer, which is efficient for tree-ensemble models. For interpretability only, XGBoost was tuned and refit on the full processed descriptor matrix, and SHAP values were computed on a fixed random subsample of 250 repeat units to reduce computational cost while approximating global attribution patterns. Global importance was summarized as the mean absolute SHAP value per descriptor. These attributions were interpreted as descriptor-level model associations rather than causal physical explanations.

For SVR, model-agnostic permutation feature importance was computed using sklearn.inspection.permutation_importance. Hyperparameters were selected using Optuna, and the final SVR model was refit on the full processed descriptor matrix. Permutation importance was then evaluated on the same fixed random subsample of 250 repeat units used for the XGBoost SHAP analysis, with the subsample selected using random seed = 123. For each feature, values in the subsample were randomly permuted 30 times while all other features were held fixed. The importance was reported as the mean increase in RMSE after permutation, with standard deviation across repeats. Features were ranked by the mean RMSE increase.

This descriptor-importance analysis was interpretive and was not averaged over cross-validation folds or random seeds. Because SHAP and permutation importance quantify different model-specific attribution concepts, their magnitudes were not directly compared; instead, results were interpreted at the level of broad descriptor families.

### 2.12 Evaluation Metrics and Paired Statistical Comparisons

Model accuracy was quantified using three standard regression metrics computed on the outer test folds for each validation regime, fold, seed, and training fraction: root mean squared error (RMSE), mean absolute error (MAE) and the coefficient of determination (*R* ^2^). RMSE and MAE report error magnitude in Kelvin, while *R* ^2^ measures variance explained relative to the test-set mean. These metrics are expressed as follows (eq 5).

5 $$RMSE=\sqrt{\frac{1}{n}\sum_{i=1}^{n}{\left(y_{i}-{ŷ}_{i}\right)}^{2}},,MAE=\frac{1}{n}\sum_{i=1}^{n}\left|y_{i}-{ŷ}_{i}\right|,,R^{2}=1-\frac{\sum_{i=1}^{n}{\left(y_{i}-{ŷ}_{i}\right)}^{2}}{\sum_{i=1}^{n}{\left(y_{i}-{\mathrm{y̅}}_{i}\right)}^{2}}$$

For paired model comparisons, SVR and XGBoost were compared across matched seed/fold evaluations within each validation regime. Because the number of paired evaluations was limited and normality was not assumed, paired Wilcoxon signed-rank tests were used as nonparametric descriptive comparisons. Paired mean differences, calculated as SVR—XGBoost, and bootstrap confidence intervals were reported to quantify effect size and uncertainty. These statistical comparisons were applied to the main regression metrics and to conformal mean interval-width at α = 0.10. For RMSE, MAE, and conformal mean interval width, negative differences indicate lower values for SVR; for *R* ^2^, positive differences indicate higher values for SVR. Because repeated cross-validation evaluations are not fully independent and multiple metrics/regimes were examined, these comparisons were interpreted conservatively as benchmark-specific descriptive evidence, not proof of general algorithm-class superiority.

### 2.13 Screening, Ranking, and Interval-Aware Triage Analysis

Candidate screening in materials discovery is often driven by ranking quality and high-performing subset selection, not solely by global regression error. Therefore, we performed a retrospective screening-utility analysis using outer-test predictions. For each outer test fold, candidate polymers were evaluated under *T*_g_-targeted selection rules using *T*_g,target_ thresholds of 300, 350, and 400 K. Ranking utility was quantified using Spearman rank correlation, concordance index, Precision@k, Recall@k, enrichment factor, and false-positive rate for high-*T*_g_ candidate selection. Precision@k and Recall@k were computed using the top 10% of the candidates within each outer test fold. These metrics were used to evaluate candidate-selection utility, specifically whether the models could enrich high-*T*_g_ polymers among top-ranked candidates, not merely achieve low global prediction error.

To evaluate uncertainty-aware triage, we compared three decision rules using conformal intervals at α = 0.10. First, point-prediction selection classified a candidate as positive when $ŷ\geq T_{g,target}$ . Second, conservative lower-bound selection classified a candidate as confidently positive only when the lower conformal bound exceeded the target: $ŷ-{\mathrm{q̂}}_{a}\geq T_{g,target}$ Third, uncertainty-flagged triage marked candidates as inconclusive when their conformal intervals overlapped *T*_g,target_. Under this rule, candidates with intervals entirely above the target were considered confidently positive, candidates with intervals entirely below the target were considered confidently negative, and threshold-overlapping intervals were abstained from decision-making. False-positive rate was defined as the fraction of truly below-threshold candidates incorrectly selected as positives. We report selected fraction, precision, recall, false-positive rate, enrichment factor, and uncertainty-flagging or abstention rate. This analysis is interpreted as a retrospective decision-utility diagnostic, not as evidence of prospective experimental screening performance.

## 3 Results and Discussion

The goal of this benchmark is not to propose a new predictive model, but to evaluate how established descriptor-based models and UQ tools behave under validation regimes imposing different forms of train-test separation in a small polymer *T*_g_ data set.

### 3.1 Quantifying The Generalization Gap across Validation Regimes

We evaluated SVR and XGBoost under validation regimes imposing different forms of train-test separation, moving from stratified/interpolative splitting to scaffold-based and similarity-clustered splits. All results were computed using 5-fold cross-validation repeated over three random seeds, and Table 2 summarizes performance at full training fraction (frac = 1.0) across all regimes. As split constraints reduce train-test structural support, performance generally degrades and variability increases, most strongly under scaffold splitting, revealing a regime-dependent generalization gap that is not apparent under stratified evaluation alone (Table 2; Figure 1).

**Table 2:** Predictive Performance of SVR and XGBoost for Polymer Glass Transition Temperature (*T*_g_) Under Stratified, Scaffold-Based, and Fingerprint-Clustered Splitting Regimes at Full Training Fraction (Frac = 1.0)^a^

regime	model	RMSE	*R* ^2^	MAE
cluster (*c* = 0.20)	SVR	36.078 ± 20.391	0.535 ± 0.278	21.65 ± 5.269
cluster (*c* = 0.20)	XGBoost	40.435 ± 21.631	0.385 ± 0.382	23.446 ± 4.96
cluster (*c* = 0.30)	SVR	32.938 ± 13.845	0.621 ± 0.222	19.894 ± 2.357
cluster (*c* = 0.30)	XGBoost	38.051 ± 15.684	0.47 ± 0.353	21.658 ± 2.778
cluster (*c* = 0.40)	SVR	33.056 ± 13.380	0.625 ± 0.191	20.444 ± 3.266
cluster (*c* = 0.40)	XGBoost	34.662 ± 14.229	0.573 ± 0.241	21.024 ± 3.239
scaffold	SVR	32.355 ± 13.082	0.523 ± 0.207	21.978 ± 5.222
scaffold	XGBoost	34.904 ± 16.532	0.367 ± 0.595	21.206 ± 4.002
stratified	SVR	32.774 ± 14.54	0.635 ± 0.219	19.437 ± 2.838
stratified	XGBoost	35.368 ± 15.23	0.563 ± 0.277	20.567 ± 1.855

a Values are reported as mean ± standard deviation across 15 outer-test evaluations (5-fold cross-validation x 3 random seeds; seeds 42/43/44). Metrics include RMSE (K), MAE (K) and *R* ^2^_._

**Figure 1:**
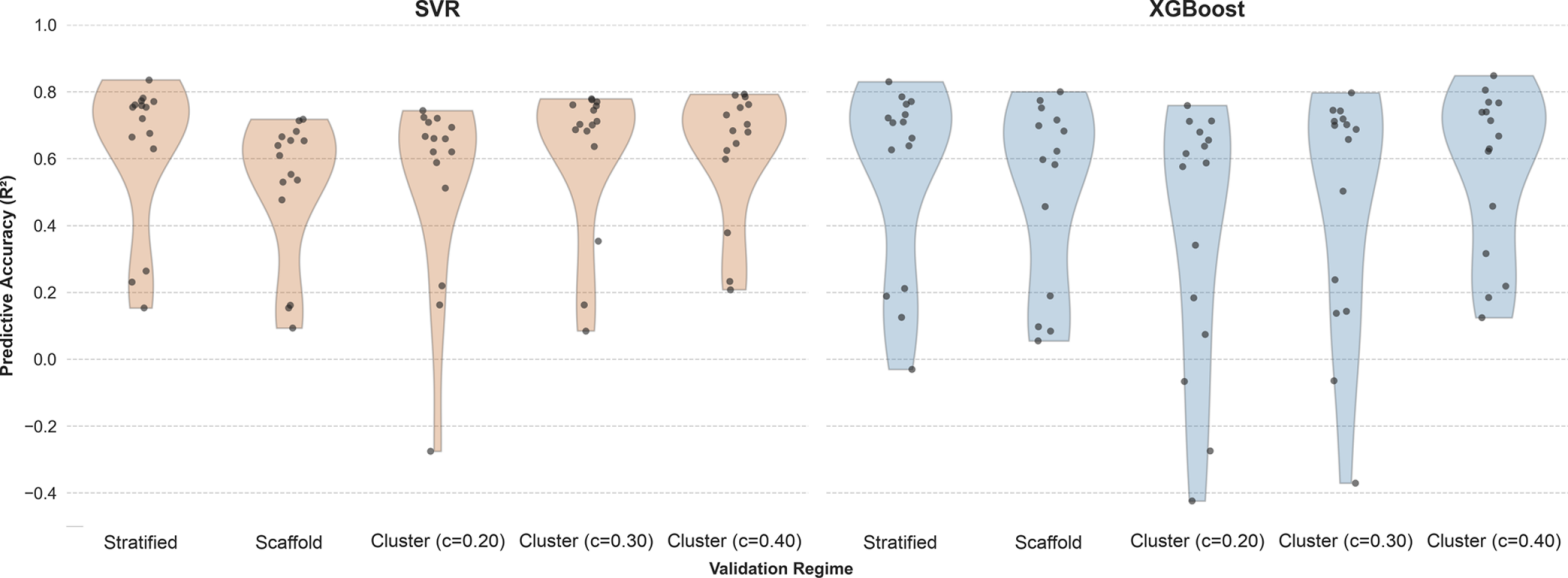
Generalization gap across validation regimes for polymer *T*_g_ prediction. Distribution of outer test *R* ^2^ scores for SVR (left) and XGBoost (right) under stratified (interpolative), scaffold-based, and fingerprint-clustered splitting regimes (*c* = 0.20, 0.30, 0.40). Each point represents one outer-fold evaluation (5 folds × 3 random seeds; *n* = 15 per regime), and violins show the corresponding performance distributions.

The observed generalization gap may partly reflect limitations of the feature representation. Under stratified splitting, chemically similar repeat units in the training set may compensate for missing macromolecular information through interpolation. Under scaffold- or cluster-based splitting, however, test polymers are less supported by local chemical analogues, and omitted variables such as molecular weight, tacticity, branching, packing, morphology, or protocol-dependent structural organization may become more consequential. Therefore, the generalization gap should be interpreted as the combined effect of train-test chemical shift, small sample size, model bias, and representation insufficiency, rather than as a purely algorithmic effect.

Under the stratified regime, both models achieved strong in-distribution accuracy, indicating that the descriptor representation captures structure–property signal for *T*_g_. The SVR model reached a best-fold *R* ^2^ of 0.836 (MAE = 15.48 K), while XGBoost reached a best-fold *R* ^2^ of 0.829 (MAE = 17.47 K). Importantly, these peak values occur under an evaluation setting with substantial structural overlap between train and test folds and therefore represent interpolative performance rather than robustness under novelty (Table 2; Figure 1).

Because the standard deviations of SVR and XGBoost performance overlapped in several regimes, paired statistical comparisons were performed across matched seed/fold evaluations. These comparisons are reported in Table S5. The results support lower point-prediction error for SVR in the pooled analysis and in the cluster *c* = 0.20 and *c* = 0.30 regimes. However, differences were weaker or not statistically supported under stratified, scaffold, and cluster *c* = 0.40 settings. Therefore, SVR’s apparent advantage is interpreted as a benchmark-specific trend in point-prediction stability, not as evidence of general superiority of kernel methods over tree ensembles.

When the split enforces greater novelty, the generalization gap becomes more pronounced. Under scaffold splitting, SVR showed numerically higher mean performance (*R* ^2^ = 0.523 ± 0.212), whereas XGBoost exhibited lower mean performance with larger variability (*R* ^2^ = 0.367 ± 0.609), consistent with fold-to-fold instability visible as extended lower tails in the regime-wise *R* ^2^ distributions (Figure 1). In this benchmark, SVR showed more stable average point-prediction performance than XGBoost under several novelty-enforcing regimes. We interpret this result as an empirical observation specific to the present data set, descriptor representation, tuning budget, preprocessing pipeline, and split design, not as a general conclusion about kernel methods versus tree ensembles in polymer informatics. The smoothness and regularization properties of RBF-kernel SVR may partly contribute to this behavior, but this explanation remains speculative. The observed XGBoost variability may also reflect fold-specific chemistry distributions, descriptor dimensionality relative to sample size, the limited hyperparameter search budget, the larger XGBoost hyperparameter space, regularization settings, or residual imbalance introduced by nonrandom splits.

We caution that scaffold splitting should be interpreted as a framework-leakage diagnostic rather than a definitive polymer-specific novelty criterion. The scaffold characterization (Table S2) shows that 73.7% of repeat units are ring-containing and can therefore be assigned nonempty Murcko frameworks, supporting the use of scaffold splitting as an intermediate framework-level diagnostic for this subset. However, 26.3% of repeat units are acyclic and return an empty scaffold, and 79.3% of scaffold groups are singletons. These results indicate that Murcko scaffold labels are informative for part of the data set but incomplete as a general polymer novelty criterion. Therefore, the effective difficulty of scaffold splitting was assessed empirically through *S*_max_ distributions and scaffold-characterization statistics rather than assumed from the Murcko definition alone.

To characterize the effective difficulty of the cluster-based splits, split-diagnostics were used to report cluster counts, cluster-size distributions, singleton-cluster fractions, fold-size balance, fold-level *T*_g_ summaries, chemistry-summary statistics, *S*_max_ distributions, and low-*S*_max_ tail fractions (Table S3; Figure S1). The cutoffs generated distinct cluster granularities: *c* = 0.20 produced fewer and broader clusters, including the largest cluster of 118 repeat units, whereas *c* = 0.40 produced many smaller clusters and a higher singleton fraction. However, median *S*_max_ values remained relatively close across cutoffs, indicating that these regimes should not be interpreted as a sharply monotonic OOD hierarchy. Instead, the low-*S*_max_ tail fraction provides a more informative view of the subset of test polymers with the weakest training-set support, with *c* = 0.20 producing the largest low-similarity tail.

To evaluate whether the cluster-split characterization was specific to the default Morgan fingerprint configuration, we repeated the split-diagnostic analysis using alternative Morgan fingerprint settings. As shown in Figure S2, absolute cluster counts, singleton-cluster fractions, and low-*S*_max_ tail fractions were sensitive to the fingerprint configuration, particularly when using radius 3/2048-bit fingerprints. However, the qualitative cutoff-dependent trends were preserved: lower cutoffs produced broader clusters and larger low-similarity tails, whereas higher cutoffs produced more fragmented clusters and higher singleton fractions. Therefore, the selected cutoffs are interpreted as operational similarity-based stress test settings rather than universal polymer OOD benchmarks. This sensitivity analysis characterizes split-behavior only; it does not establish model-performance robustness across all possible fingerprints or clustering algorithms.

Consistent with the split-characterization analysis, the cluster *c* = 0.20 split produced the largest low-*S*_max_ tail and was therefore treated as the strongest cluster-based stress test among the examined settings. Under this regime, XGBoost showed its largest performance degradation (*R* ^2^ = 0.385 ± 0.391; MAE = 23.45 ± 5.08 K), while SVR remained comparatively more stable (*R* ^2^ = 0.535 ± 0.284; MAE = 21.65 ± 5.39 K). Overall, this observation is interpreted as a benchmark-specific stability trend, not as a general conclusion about kernel-based methods versus tree ensembles in polymer *T*_g_ prediction.

The present benchmark is limited to two classical descriptor-based regressors. Recent polymer ML studies increasingly use richer architectures and representations, including graph neural networks,^49^ directed message-passing neural networks,^50^ polymer-specific graph representations,^51^ multimodal polymer representations,^7^ pretrained models,^52,53^ and uncertainty-aware deep learning methods.^26^ These approaches may improve accuracy or representation quality relative to classical descriptor-based models, particularly when larger data sets or richer structural annotations are available. Therefore, the present results should be interpreted as a reliability analysis for two classical small-data baselines, not as a comprehensive comparison against the current frontier of polymer ML.

### 3.2 Sample Efficiency under Increasing Novelty

To quantify how data availability interacts with validation difficulty, we constructed learning curves across training fractions (0.2–1.0) for both models and each validation regime. Under stratified splitting, both models improved systematically with additional data and exhibited diminishing returns beyond 0.8 training fraction, consistent with saturation once the dominant in-distribution signal was learned. Increasing the training fraction from 0.2 to 1.0 reduced mean RMSE by 3.48 K for SVR and 3.26 K for XGBoost, with much of the improvement occurring before the 0.8 fraction. Across regimes, SVR reached near-asymptotic performance at smaller training fractions than XGBoost, suggesting greater sample efficiency within the data-sparse setting studied here (Figure 2).

**Figure 2:**
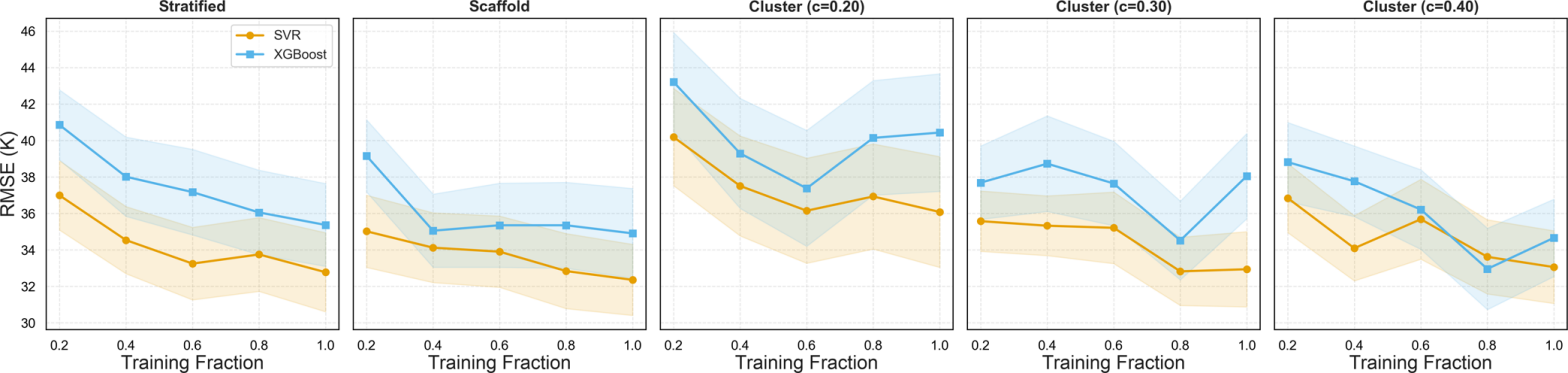
Learning curves (sample efficiency) under increasing chemical novelty. Mean outer-test RMSE (K) for SVR and XGBoost as a function of training fraction (0.2–1.0) under stratified (interpolative), scaffold-based, and fingerprint-clustered splitting regimes (*c* = 0.20, 0.30, 0.40). Shaded bands indicate ±1 standard deviation, capturing uncertainty arising from fold/seed variability.

Scaffold-based splitting showed an intermediate pattern: both models improved from the lowest training fraction, but the curves remained less smooth than under stratified splitting, consistent with reduced framework-level support and greater fold-to-fold variability.

In contrast, under cluster-based splits (*c* = 0.20, 0.30, and 0.40), RMSE reductions were smaller and accompanied by larger fold-to-fold variability. Neither model showed clear saturation at the maximum training fraction, consistent with a novelty-limited regime in which additional samples are most beneficial when they expand coverage of chemically relevant neighborhoods. Accordingly, the learning curves emphasize that data quantity and domain shift interact: increasing sample size improves interpolative performance reliably, whereas gains under novelty depend on chemical coverage rather than sample count alone (Figure 2, Table S6). Full run level results and conformal outputs are provided in Table S6, with column definitions in Table S7.

### 3.3 Applicability Domain and Chemical Similarity Analysis

To characterize structural support as one component of AD, we computed, for each test polymer, the maximum Morgan-Tanimoto similarity to the corresponding training set (*S*_max_; radius = 2, 2048 bit). *S*_max_ serves as an operational proxy for structural support from training chemistry, where lower *S*_max_ values indicate greater extrapolation demand (weaker structural support). The resulting *S*_max_ distributions show that nearest-neighbor structural support differs across evaluation regimes (Figures 3, 4). However, because *S*_max_ captures only one component of AD, we interpret these distributions as structural-similarity diagnostics rather than complete measures of deployment risk.

**Figure 3:**
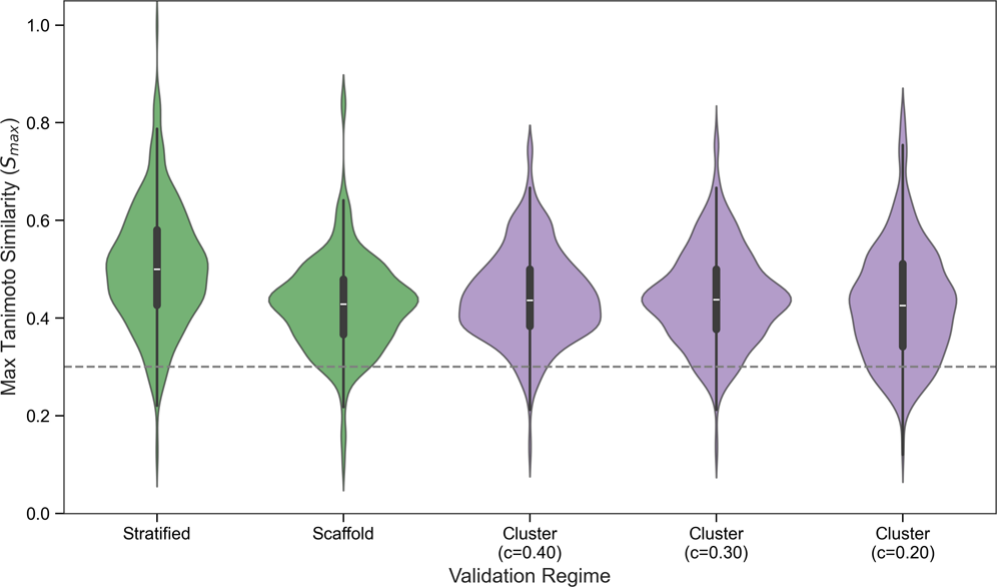
Applicability-domain diagnostic via maximum test-to-train similarity across validation regimes. Violin plots show the distribution of the maximum Morgan-Tanimoto similarity *S*_max_ (radius = 2, 2048-bit fingerprints) between each test polymer and its corresponding training set, computed for every outer-fold evaluation.

**Figure 4:**
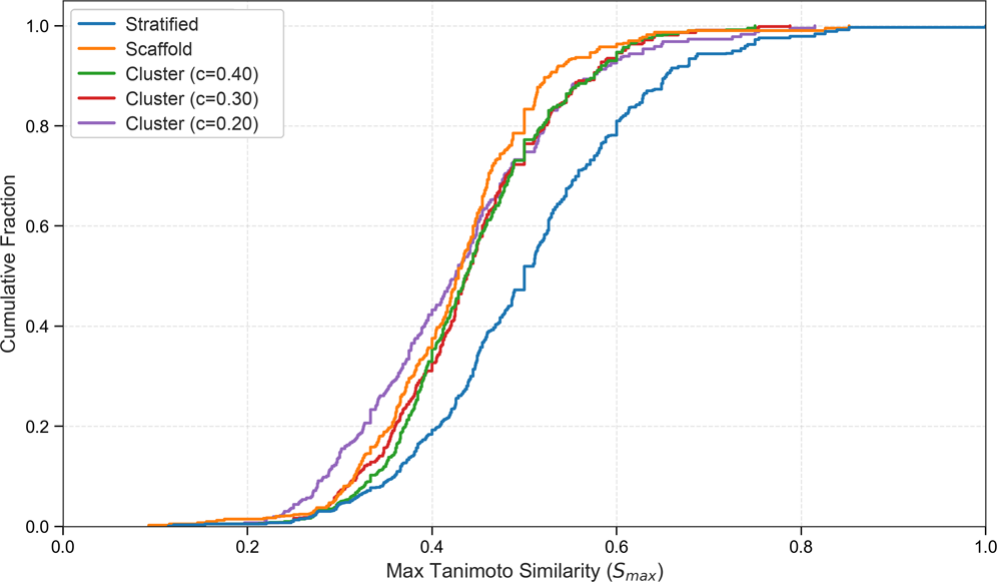
Cumulative distributions of maximum test-to-train similarity quantify novelty across split regimes. Empirical cumulative distribution functions (ECDFs) of the maximum Morgan-Tanimoto similarity *S*_max_ between each test polymer and its corresponding training set (Morgan fingerprints, radius = 2, 2048 bits), aggregated over all outer-fold evaluations.

Under stratified splitting, the *S*_max_ distribution was narrow and unimodal (centered at 0.45–0.47), consistent with the fact that property-balanced random partitioning retains substantial structural overlap between folds in fingerprint space.^54^ Notably, scaffold splitting produced broader distributions (0.35–0.55), reflecting the fact that Murcko-disjoint cores can still share local substructures that remain similar under Morgan fingerprints.^20,55^ This similarity analysis is important because scaffold identity alone does not fully define chemical novelty in polymer repeat units. Distinct Murcko scaffolds may still share local chemical environments, while flexible or acyclic repeat units may yield weakly informative scaffold labels. Therefore, *S*_max_ is used as an independent post hoc diagnostic to quantify the actual train-test similarity induced by each splitting regime. The scaffold statistics (Table S2) support the use of scaffold splitting as an intermediate framework-level leakage-control diagnostic, while also confirming that scaffold identity should be interpreted together with fingerprint-based *S*_max_ diagnostics rather than as a complete measure of polymer novelty.

Across cluster-based regimes, the distributions systematically reflected the chosen cutoffs. The most stringent regime, cluster (*c* = 0.20), exhibited the lowest central tendency (mean ∼0.43) and the broadest tail toward low similarity (Figure 3). In comparison, cluster (*c* = 0.30) and cluster (*c* = 0.40) regimes were shifted rightward (means ∼ 0.44–0.45) and displayed comparatively weaker low-similarity tails. All cluster-based splits retained a distinct fraction of low-similarity test instances (maximum similarity ∼0.1–0.2), confirming the intended separation of distinct chemical neighborhoods.

The distribution profiles indicate that cluster *c* = 0.20 produced the lowest nearest-neighbor structural support among the cluster-based splits considered here. However, the median *S*_max_ values remained moderate and relatively close across regimes, indicating that these splits primarily probe mild-to-moderate novelty rather than extreme extrapolation. Because median *S*_max_ values alone can obscure differences in the low-similarity region of the test distribution, we additionally examined low-*S*_max_ tail fractions. The cluster *c* = 0.20 split produced the largest fraction of test predictions with *S*_max_ < 0.30, supporting its interpretation as the strongest cluster-based stress test among the settings examined with respect to low-similarity tail enrichment. However, this does not imply that the cluster cutoffs define a universal or fully realistic polymer deployment hierarchy.

We then linked novelty to predictive reliability by examining error as a function of *S*_max_. A novelty-related error trend was observed, with larger errors concentrated among lower-*S*_max_ test polymers (Figure 5A). Consistent with this trend, conformal prediction intervals are wider under novelty-enforcing evaluation regimes, indicating an uncertainty response aligned with extrapolative risk (Figure 5B).

**Figure 5:**
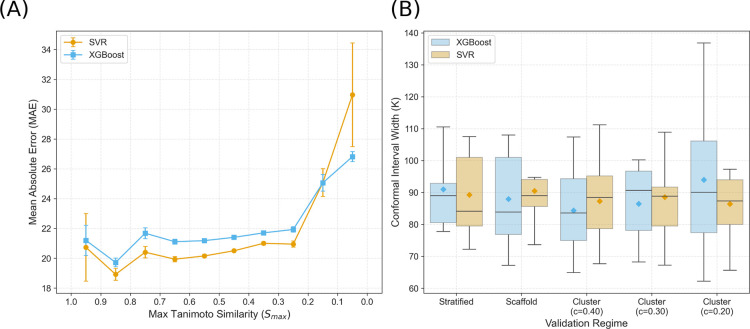
Novelty penalty and uncertainty response under chemical shift. (A) Relationship between structural proximity and point-error: mean absolute error (MAE, K) for SVR and XGBoost plotted against binned maximum test-to-train similarity *S*_max_ (Morgan fingerprints, radius = 2, 2048 bits), showing increasing error as similarity decreases. Points denote bin-wise means and error bars indicate variability across outer-fold evaluations. (B) Distribution of split conformal prediction interval widths across validation regimes. Box plots summarize interval-width distributions (median and interquartile range; whiskers indicate spread), with diamonds marking means.

#### 3.3.1 Expanded Applicability-Domain Diagnostics

Maximum fingerprint similarity, *S*_max_, provides a useful first-order measure of nearest-neighbor structural support, but it does not fully capture descriptor-space sparsity or target-space support. Therefore, we evaluated complementary AD diagnostics, including k-nearest-neighbor similarity, Tanimoto-neighborhood density, standardized descriptor nearest-neighbor distance, PCA-space distance, and retrospective target-space support; these expanded diagnostics are summarized in Figure S3 and Tables S8 and S9.

The multimetric analysis showed that the different AD diagnostics capture related but nonidentical aspects of prediction support. As expected, *S*_max_ was strongly associated with kNN similarity, whereas descriptor nearest-neighbor distance, PCA-space distance, and retrospective target-space diagnostics provided additional information not fully represented by maximum fingerprint similarity. These results support interpreting *S*_max_ as a structural-similarity diagnostic rather than as a standalone AD measure.

Threshold-based AD analysis further showed that outside-AD predictions tended to have higher MAE and lower conformal coverage at α = 0.10 than inside-AD predictions. This trend was observed for low-*S*_max_ samples, low-kNN-similarity samples, descriptor-distance outliers, and PCA-distance outliers. Composite structural outside-AD predictions also showed higher error and lower coverage than predictions satisfying all structural AD criteria. These findings support the use of multiple AD diagnostics to identify higher-risk predictions, while retrospective target-space diagnostics are interpreted only as a post hoc error analysis, not as prospective screening criteria.

### 3.4 Conformal Prediction and Uncertainty Quantification

To move beyond point estimates toward decision-support outputs, we applied SCP to both SVR and XGBoost. Within each outer training fold, we reserved 20% of the training data for calibration, defined nonconformity scores as absolute residuals, and constructed symmetric prediction intervals using the (1-α) quantile of calibration residuals. We evaluated the coverage-width trade-off by varying the miscoverage level (α) across 0.05, 0.10 and 0.20, corresponding to nominal confidence levels of 95%, 90% and 80%, respectively.

Across regimes and α-levels, empirical coverage aligned closely with the nominal confidence levels at the marginal level for both models (Table 3). However, marginal validity does not guarantee uniform reliability across chemical space, particularly under chemical shift. Therefore, subgroup-level coverage was evaluated separately as a local reliability diagnostic, as discussed in Section 3.5. At α = 0.05, empirical coverage ranged from 0.942 to 0.961 across regimes and models, including novelty-enforcing settings where point accuracy declined, such as cluster *c* = 0.20. These results are consistent with SCP’s marginal validity and indicate that, in our evaluation, internal calibration remained close to nominal across regimes. Because SCP provides marginal rather than conditional coverage, reliability within specific chemical subregions may still vary under distribution shift.^56,57^

**Table 3:** Empirical Coverage and Mean Interval Width of Split Conformal Prediction Intervals for SVR and XGBoost across Validation Regimes at Miscoverage Levels α = 0.20, 0.10, and 0.05 (Nominal Coverages 80%, 90%, and 95%)^a^

regime	model	alpha	cov mean	width mean (K)	target coverage
cluster (*c* = 0.20)	SVR	0.2	0.801	60.809	0.8
cluster (*c* = 0.20)	SVR	0.1	0.886	84.327	0.9
cluster (*c* = 0.20)	SVR	0.05	0.947	119.390	0.95
cluster (*c* = 0.20)	XGBoost	0.2	0.778	60.914	0.8
cluster (*c* = 0.20)	XGBoost	0.1	0.884	92.231	0.9
cluster (*c* = 0.20)	XGBoost	0.05	0.947	133.377	0.95
cluster (*c* = 0.30)	SVR	0.2	0.816	62.776	0.8
cluster (*c* = 0.30)	SVR	0.1	0.910	88.546	0.9
cluster (*c* = 0.30)	SVR	0.05	0.955	118.272	0.95
cluster (*c* = 0.30)	XGBoost	0.2	0.802	61.331	0.8
cluster (*c* = 0.30)	XGBoost	0.1	0.906	86.478	0.9
cluster (*c* = 0.30)	XGBoost	0.05	0.961	119.966	0.95
cluster (*c* = 0.40)	SVR	0.2	0.779	58.046	0.8
cluster (*c* = 0.40)	SVR	0.1	0.897	87.282	0.9
cluster (*c* = 0.40)	SVR	0.05	0.947	115.859	0.95
cluster (*c* = 0.40)	XGBoost	0.2	0.771	58.762	0.8
cluster (*c* = 0.40)	XGBoost	0.1	0.901	84.373	0.9
cluster (*c* = 0.40)	XGBoost	0.05	0.956	122.488	0.95
scaffold	SVR	0.2	0.751	61.436	0.8
scaffold	SVR	0.1	0.888	90.821	0.9
scaffold	SVR	0.05	0.943	118.422	0.95
scaffold	XGBoost	0.2	0.792	61.221	0.8
scaffold	XGBoost	0.1	0.897	89.074	0.9
scaffold	XGBoost	0.05	0.952	116.509	0.95
stratified	SVR	0.2	0.818	60.975	0.8
stratified	SVR	0.1	0.917	89.273	0.9
stratified	SVR	0.05	0.955	115.045	0.95
stratified	XGBoost	0.2	0.816	62.092	0.8
stratified	XGBoost	0.1	0.918	90.964	0.9
stratified	XGBoost	0.05	0.952	117.545	0.95

a Cov mean reports the fraction of outer-test points contained within the conformal interval, and width mean reports the average interval width (K). Values are aggregated over the 15 outer-test evaluations (5 folds × 3 seeds), demonstrating near-nominal coverage across both interpolative (stratified) and novelty enforcing (scaffold/cluster) settings, as expected for split conformal prediction under the exchangeability assumption.

Interval efficiency, quantified by mean interval width (MIW), varied by regime and model (Figure 6). As expected, relaxing the coverage level (increasing α) reduced MIW. Under the stratified splitting, SVR achieved MIWs of 115.0 K (α = 0.05), 89.3 K (α = 0.10), and 61.0 K (α = 0.20), while XGBoost required slightly wider intervals (117.5, 91.0, and 62.1 K, respectively) to achieve comparable coverage. Under novelty-enforcing splits, XGBoost exhibited larger variability in MIWs than SVR, (e.g., SD = 57.9 K at α = 0.05 under cluster *c* = 0.20 versus 25.6 K for SVR), consistent with more heterogeneous residual behavior under stronger extrapolation pressure (Figure 6). Scaffold splitting likewise preserved near-nominal coverage (∼94–95% at α = 0.05) while modestly increasing MIW, underscoring that uncertainty estimates remain useable when test chemistry departs from the training domain.

**Figure 6:**
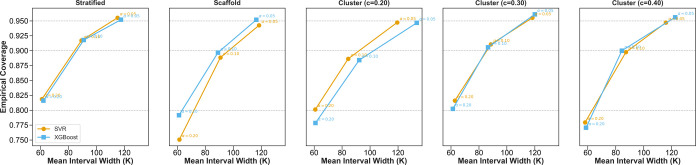
Conformal uncertainty quantification: coverage-width trade-off across regimes (α-sweep). Empirical coverage (*y*-axis) versus mean prediction-interval width (*x*-axis) for split conformal prediction applied to SVR and XGBoost under each validation regime (stratified, scaffold, and fingerprint-cluster splits with *c* = 0.20/0.30/0.40). Points correspond to miscoverage levels α = 0.20, 0.10, and 0.05 (nominal coverages 80%, 90%, and 95%), connected to visualize the trade-off between tighter intervals and higher coverage.

We also compared SVR and XGBoost conformal MIWs at α = 0.10 using paired tests across matched seed/fold evaluations. These comparisons are reported in Table S5 and are interpreted as descriptive evidence of interval-efficiency differences within this benchmark. Although empirical coverage remained close to the nominal levels, this should not be interpreted as sufficient evidence of practical decision utility. The 95% conformal intervals are relatively wide; for example, SVR produced MIWs of 115.0 K under stratified splitting and 119.4 K under cluster *c* = 0.20, while XGBoost reached 133.4 K under cluster *c* = 0.20. These widths limit the use of 95% intervals for fine-grained ranking of candidates with similar predicted *T*_g_ values. We therefore interpret the intervals primarily as a risk-assessment layer: they identify predictions whose uncertainty is large enough to warrant caution, additional simulation, or experimental validation, not as standalone ranking tools.

A limitation of the standard absolute-residual split conformal implementation used here is that interval half-widths are constant within each calibration fold and model/α setting. Thus, a well-supported test polymer and a chemically novel test polymer can receive intervals of similar width, even though their expected prediction difficulty may differ. This limits the usefulness of standard split conformal intervals for individual candidate ranking. Adaptive variants, such as normalized nonconformity scores, Mondrian conformal prediction conditioned on similarity or chemistry groups, and conformalized quantile regression, may provide more informative local intervals in future work. However, such methods require sufficient calibration data within local strata to avoid unstable subgroup quantiles, which is challenging in the present small-data setting. These findings motivate the subsequent ranking and interval-aware triage analysis.

### 3.5 Subgroup Coverage and Local Reliability Diagnostics

Marginal empirical coverage remained close to nominal across models and split regimes; however, marginal coverage can obscure subgroup-level undercoverage. Therefore, we evaluated empirical coverage and mean interval width as a function of *S*_max_, *T*_g_ range, and broad repeat-unit chemistry classes. This analysis is particularly relevant for novelty-aware polymer screening because undercoverage in low-similarity, high-*T*_g_, or chemistry-specific subsets would indicate that the intervals may be less reliable in regions where extrapolative risk is expected to be higher.

The subgroup coverage results are summarized in Table S10 and visualized in Figure S4. At the 90% nominal level (α = 0.10), low-*S*_max_ polymers showed undercoverage for both SVR and XGBoost, whereas medium- and high-*S*_max_ groups were close to or above nominal coverage. Coverage also tended to be lower in high-*T*_g_, acyclic, and heteroatom-rich subsets. These findings indicate that marginal coverage can overstate reliability for specific subgroups. Accordingly, the subgroup results are used to contextualize marginal coverage and identify local reliability limitations, not to establish formal conditional-coverage guarantees.

### 3.6 Screening, Ranking, and Interval-Aware Triage Utility

Regression metrics provide a global summary of prediction error, but they do not directly measure whether a model can prioritize high-*T*_g_ candidates for screening. We therefore evaluated ranking and threshold-based triage performance under *T*_g_-targeted selection rules at 300, 350, and 400 K. This analysis separates absolute regression performance from candidate-selection utility and tests whether conformal intervals reduce overconfident false-positive selection.

Point-prediction ranking was used to evaluate whether the models enriched high-*T*_g_ polymers among the top-ranked candidates. In parallel, conformal lower-bound selection at α = 0.10 was used as a conservative triage rule, where a candidate was considered confidently above the target only when the lower bound of its conformal interval exceeded the target *T*_g_. Candidates whose intervals overlapped the threshold were treated as inconclusive, not confidently selected or rejected.

The results are reported in Tables S11 and S12 and Figures S5 and S6. Ranking metrics showed that both models retained useful nonrandom ordering ability across several validation regimes. However, the interval-aware triage analysis revealed a clear precision–recall trade-off. Conservative lower-bound selection reduced false-positive rates, particularly at 300 and 350 K thresholds, but substantially reduced recall and increased the fraction of inconclusive candidates. At the stringent 400 K threshold, both models produced few or no confident selections. Therefore, conformal intervals are interpreted here as conservative triage tools, not as high-resolution screening instruments.

### 3.7 Model-Specific Descriptor-Importance Analysis: XGBoost SHAP and SVR Permutation Importance

We used model-specific descriptor-importance analyses as descriptor-level plausibility checks, not as physical validation of polymer *T*_g_ mechanisms. Because the models were trained on repeat-unit 2D descriptors and proxy variables, the resulting attributions identify how each fitted model uses the available representation rather than causal molecular determinants of *T*_g_. For XGBoost, SHAP values were computed using TreeExplainer (TreeSHAP),^58^ which is specialized for tree-ensemble models. To obtain a global descriptor-importance summary, XGBoost was retuned with Optuna and refit on the full data set using the complete descriptor matrix (RDKit 2D descriptors augmented with polymer proxy features). This refit was performed only for interpretability and was not used to report predictive performance under any validation regime. SHAP values were computed on a random subset of 250 polymers to reduce computational cost while approximating global attribution patterns, and global feature importance was summarized as the mean absolute SHAP value per descriptor (mean |SHAP|; Figure 7A).

**Figure 7:**
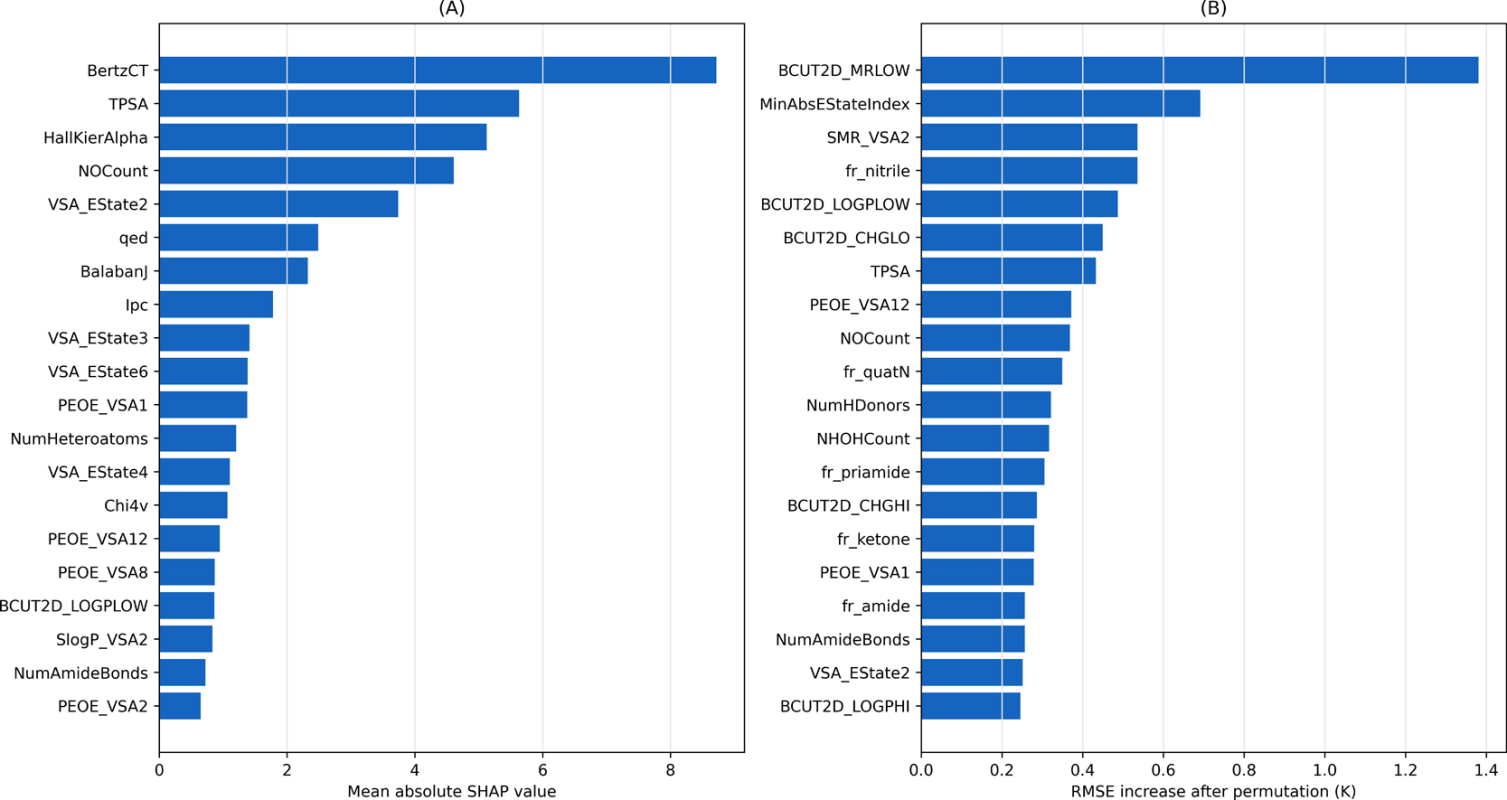
Model-specific descriptor-importance analysis. (A) XGBoost feature importance summarized by mean absolute SHAP values. (B) SVR permutation feature importance summarized as the increase in RMSE after descriptor permutation. The two panels use different model-specific importance measures, and their magnitudes are not directly comparable. The full rank comparison and SVR permutation-importance standard deviations are provided in Table S13.

Because many 2D molecular descriptors are correlated, attribution mass may be redistributed among related variables, and feature rankings may not be unique. We therefore interpret SHAP and permutation-importance results at the level of broad descriptor families rather than as uniquely resolved contributions of individual descriptors. The descriptor correlation heatmap is used to contextualize these attribution patterns (Figure S7).

To align descriptor-importance analysis with the model-comparison results, we additionally evaluated SVR using permutation feature importance. As shown in Figure 7B and Table S13, SVR importance rankings differed from XGBoost SHAP rankings at the individual-feature level, indicating that descriptor reliance is model-specific. This is expected because SHAP and permutation importance quantify different attribution concepts and were not compared by magnitude. Nevertheless, both analyses highlighted chemically plausible descriptor families related to polarity/surface area, heteroatom or functional-group content, and electronic-state descriptors. Therefore, these results are interpreted as model-level descriptor-reliance diagnostics rather than as evidence of unique physical determinants of *T*_g_.

For XGBoost, the global ranking highlighted BertzCT, TPSA, HallKierAlpha, NOCount, and several VSA/EState descriptors as dominant contributors to predicted *T*_g_ (Figure 7A; Table S13). These descriptors are associated with repeat-unit complexity, topology, polarity, surface-area patterns, and N/O heteroatom content within the adopted feature space. Their appearance is chemically plausible for *T*_g_ prediction because local rigidity, polarity, and steric features can influence segmental mobility. However, these attributions should not be interpreted as evidence that the model has recovered a complete or causal physical description of glass-transition behavior. The descriptors do not explicitly encode molecular weight, tacticity, branching, cross-linking, packing, morphology, or processing history. Therefore, the observed associations are interpreted as data set-specific relationships between repeat-unit descriptors and MD-derived *T*_g_ labels. For example, higher contributions from BertzCT (mean |SHAP| = 8.72) and HallKierAlpha (mean |SHAP| = 5.12) are consistent with the expectation that, within this data set, topological complexity and shape descriptors correlate with repeat-unit rigidity, which has been associated with elevated *T*_g_ in long-chain polymers.^59–61^ TPSA (mean |SHAP| = 5.63) shows largely positive contributions at moderate-to-high values, consistent with the documented association between polar functionality and restricted segmental mobility,^62–64^ though we note that the model cannot distinguish between different physical mechanisms through which polarity may affect *T*_g*.*_ NOCount (mean |SHAP| = 4.61), a coarse proxy for N/O heteroatom content, likely aggregates functional contexts with opposing effects (e.g., heteroatoms in rigid polar motifs versus flexible polar linkages); accordingly, it is interpreted alongside polarity and topology-related descriptors (e.g., TPSA and HallKierAlpha), rather than as a standalone variable.^65–68^

Beyond the top-ranked drivers, the appearance of multiple VSA/EState terms^69,70^ suggests that *T*_g_-relevant information is distributed across surface-area and electronic-state patterns rather than encoded by a single motif, consistent with multifactorial control of segmental dynamics.^16,71^

To connect global rankings to the learned structure–property patterns, we examined SHAP dependence plots for the most influential descriptors (Figure 8). The dependence patterns show substantial nonlinearity and interaction effects, consistent with *T*_g_ being controlled by coupled contributions from complexity/rigidity proxies, polarity, and topology. BertzCT transitions from negative to positive SHAP values as complexity increases (Figure 8A), indicating that higher complexity repeat units shift predictions toward higher *T*_g_ with saturation at the upper range. TPSA displays a threshold-like pattern (Figure 8B), in which low TPSA contributes negatively and moderate-to-high TPSA contributes positively. NOCount shows more positive contributions at higher values (Figure 8C), with interaction coloring indicating stronger effects when heteroatoms coincide with higher TPSA. HallKierAlpha exhibits a nonmonotonic pattern with clear interaction structure when colored by aromatic fraction (Figure 8D), suggesting that the model learns different topology-*T*_g_ relationships across aromatic-rich versus aromatic-poor chemistries.^72^ Finally, VSA_EState2 shows a nonlinear contribution modulated by charge-related surface area (colored by PEOE_VSA2; Figure 8E), consistent with distributed electrostatic surface patterns contributing to *T*_g_ predictions.^71^

**Figure 8:**
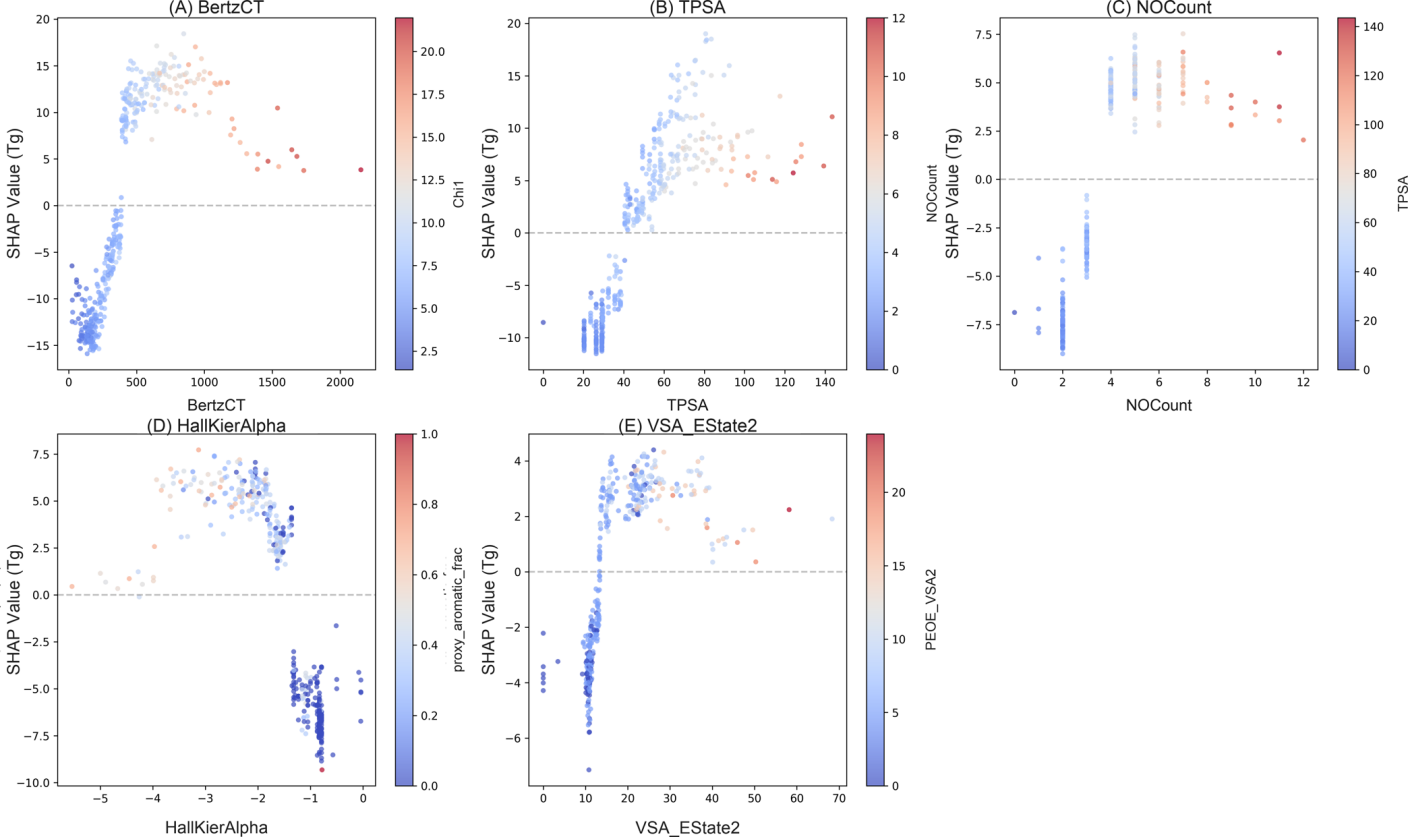
SHAP dependence plots reveal nonlinear and interaction-driven structure-*T*_g_ patterns in the XGBoost model. SHAP dependence plots for the five most influential descriptors identified by global importance (A) BertzCT, (B) TPSA, (C) NOCount, (D) HallKierAlpha, and (E) VSA_EState2. Each point corresponds to a polymer in the SHAP evaluation subset, with the *x*-axis showing the raw descriptor value and the *y*-axis showing the SHAP contribution to the predicted *T*_g_ (K). Point colors indicate an interacting feature (color bar in each panel), highlighting feature–feature dependence and nonmonotonic behavior.

Taken together, the interpretability analyses suggest that the fitted models rely on chemically plausible descriptor families, particularly polarity/surface area, heteroatom/functional-group, electronic-state, and topology-related descriptors. However, these results should be viewed as model-internal descriptor-reliance diagnostics. They support the plausibility of the learned descriptor associations but do not constitute physical validation of *T*_g_ mechanisms or proof of causal structure–property relationships.

### 3.8 Scope, Limitations, and Model-Agnostic Extension

A key limitation of the present benchmark is that the target values are MD-derived rather than experimentally measured *T*_g_ values. The results should therefore be interpreted as a study of trustworthiness with respect to a simulation-consistent label space. This distinction matters because MD-based *T*_g_ estimates are known to depend on force-field choice, cooling/heating protocol, and analysis methodology, and may differ systematically from experiment. In particular, computational cooling rates can differ substantially from experimental protocols, potentially contributing to systematic shifts between simulated and measured *T*_g_ values.^73^ Consequently, novelty-aware validation and conformal intervals in this work improve the reliability assessment of ML models trained on simulated *T*_g_ labels, but they do not by themselves establish experimental deployability.

Importantly, the reliability-assessment workflow used here is model-agnostic in design. The same novelty-aware split design, split-characterization analysis, applicability-domain diagnostics, subgroup coverage analysis, conformal prediction protocol, and interval-aware screening metrics can be applied to graph-based, multimodal, pretrained, or uncertainty-aware deep learning architectures. Such extensions would clarify whether more expressive architectures reduce the observed generalization gap, improve subgroup-level coverage, or yield more efficient and decision-useful conformal intervals under novelty-enforcing validation regimes. Therefore, the present work should be viewed as a transferable evaluation framework rather than as a final comparison of polymer *T*_g_ modeling architectures.

## 4 Conclusion

In this study, we presented a reproducible benchmark for reliability-aware polymer *T*_g_ prediction in a small, simulation-derived data set. Using validation regimes spanning stratified (interpolative), scaffold-based, and fingerprint-clustered splits, we showed that models that appear strong under interpolative evaluation can degrade under more stringent OOD conditions, indicating that weak split strategies may overstate deployable performance in data-sparse polymer informatics. Scaffold splitting should be viewed as an intermediate framework-leakage diagnostic, not as a complete polymer-specific AD criterion. Its limitations motivate the parallel use of fingerprint-clustered splits and *S*_max_-based novelty diagnostics as complementary operational stress tests. Cluster-based diagnostics showed that *c* = 0.20 produced the largest low-similarity tail; however, because median *S*_max_ values remained relatively close across cutoffs, these splits should not be interpreted as a sharply separated, monotonically ordered, or universal polymer OOD hierarchy.

Within this benchmark, SVR showed more stable point-prediction behavior than XGBoost in several novelty-enforcing split settings. However, this observation should not be generalized beyond the present data set, descriptor representation, preprocessing pipeline, and tuning protocol, and should not be interpreted as evidence of general superiority of kernel methods over tree ensembles. Learning curve analyses further showed that data availability and novelty interact: increasing training fraction improved interpolative performance more predictably, whereas gains under cluster-based splits depended more strongly on chemical coverage than on sample count alone.

SCP provided a marginally calibrated uncertainty layer across regimes, but the 95% intervals were often too wide for fine-grained candidate ranking. Ranking and interval-aware triage analyses showed that the main practical value of conformal intervals in this data set is conservative threshold-based triage: flagging uncertain predictions and reducing overconfident selections, not serving as high-resolution screening tools. Accordingly, the uncertainty estimates should be interpreted as marginally calibrated risk indicators, not as guarantees of uniformly reliable prediction across all polymer subgroups.

AD diagnostics showed that *S*_max_ is useful for identifying reduced nearest-neighbor structural support, but it captures only one component of deployment risk. Expanded diagnostics based on kNN similarity, Tanimoto-neighborhood density, descriptor-space support, PCA-space distance, target-space support, and threshold-based low-support flags provide a more cautious interpretation. Overall, the present benchmark mainly probes mild-to-moderate novelty within the data set rather than extreme extrapolation; no single AD metric should be interpreted as a complete polymer deployment-risk descriptor.

These conclusions should be interpreted within the scope of MD-derived *T*_g_ labels. While the proposed benchmark clarifies how model accuracy and uncertainty behave under novelty-aware validation in this simulated setting, direct experimental deployment will require validation against measured *T*_g_ data and, ideally, benchmarking across multiple simulation or force-field protocols. Overall, the findings should be interpreted as a reliability assessment of two classical descriptor-based baselines, not as an identification of the best polymer *T*_g_ model class. Because the present representation is limited to single-repeat-unit 2D descriptors and coarse proxy features, broader benchmarking against polymer-specific graph representations, chain-level or molecular-weight-aware descriptors, multimodal representations, pretrained models, and uncertainty-aware deep learning architectures remains an important direction for future work.

## Supplementary Material



## Data Availability

The polymer data set used in this study is provided with the code repository as data/MD_properties.csv, together with provenance information in data/README.md. The underlying source record is Project Elwood hosted by the Materials Data Facility (DOI: **10.18126/8p6m-e135**). All run level benchmarking outputs supporting the figures and tables are provided in the Supporting Information (e.g., full results table and column dictionary), enabling exact reconstruction of summary statistics reported in the main text.

## Notes

Figure S1. Fold-level *T*_g_ and chemistry distribution balance under cluster-based splitting. (A) Fold test-set size distribution across Morgan–Tanimoto cutoffs. (B) Fold-level median *T*_g_ distribution. (C) Fold-level median *S*_max_, defined as the maximum test-to-train Morgan–Tanimoto similarity. (D) Fold-level chemistry-summary distributions, including ring-containing, aromatic, and heteroatom-rich repeat-unit fractions. Boxplots summarize fold-level test-set values across repeated seed/fold partitions. Figure S2. Sensitivity of cluster-split characterization to Morgan fingerprint settings. Cluster-split diagnostics were recomputed using three Morgan fingerprint configurations: radius 2/2048 bits, used as the default setting in the main benchmark; radius 3/2048 bits; and radius 2/1024 bits. Panels show (A) number of clusters, (B) singleton-cluster fraction, (C) *S*_max_ distributions, and (D) low-*S*_max_ tail fraction, defined as the fraction of pooled test predictions with *S*_max_ < 0.30. The analysis evaluates the sensitivity of split characterization, not model performance. Figure S3. Expanded applicability-domain (AD) diagnostics. This figure summarizes relationships between prediction error and multiple AD diagnostics, including *S*_max_, kNN similarity, descriptor nearest-neighbor distance, composite inside/outside AD status, metric correlations, and outside-AD fraction by validation regime. Figure S4. Subgroup-level conformal coverage diagnostics. (A) Empirical coverage across *S*_max_ bins, *T*_g_ ranges, and broad repeat-unit chemistry classes at α = 0.10. Horizontal error bars indicate descriptive Wilson 95% confidence intervals, and the dashed vertical line indicates nominal coverage. (B) Coverage gap relative to 90% nominal coverage; negative values indicate undercoverage and positive values indicate overcoverage. (C) Mean conformal interval width across the same subgroups. Blue denotes SVR and orange denotes XGBoost. Figure S5. Ranking fidelity across validation regimes. This figure visualizes Spearman rank correlation and concordance index for SVR and XGBoost across validation regimes. Figure S6. Threshold-based conformal triage for *T*_*g*_-targeted screening. This figure visualizes precision, recall, false-positive rate, and inconclusive fraction under point-prediction selection, conservative lower-bound selection, and uncertainty-flagged triage. Figure S7. Pearson correlation heatmap for the primary SHAP-ranked descriptors. Table S1. Custom polymer proxy features included in the predictive representation. Only custom polymer proxy features included in the model are listed. These features were calculated from the processed repeat-unit SMILES using RDKit and custom RDKit-based atom-count routines. Standard RDKit molecular descriptors used in the model are not repeated in this table. Table S2. Scaffold characterization for the polymer repeat-unit data set. Murcko scaffolds were computed using the same RDKit procedure used for scaffold-based splitting. Empty scaffolds correspond to acyclic repeat units and are intentionally reported because they form a single scaffold group in the scaffold-split diagnostic. Percentages are calculated relative to the full data set unless otherwise stated in the note column. Generic Murcko scaffold abstraction is reported only as an additional diagnostic of framework compression and was not used for fold assignment. Representative examples are provided for the largest scaffold groups to illustrate the chemical meaning and limitations of scaffold-based partitioning. Table S3. Cluster-split characterization across Morgan-Tanimoto cutoffs. Cluster splits were generated using Morgan fingerprints with radius 2 and 2048 bits. *S*_max_ denotes the maximum test-to-train Tanimoto similarity. Fold-level summaries are calculated across repeated seed/fold test partitions. Low-*S*_max_ tail percentages are calculated over pooled outer-test predictions. Chemistry medians summarize fold-level test-set fractions for ring-containing, aromatic, and heteroatom-rich repeat units; heteroatom-rich repeat units were defined using the data set median heteroatom fraction. Table S4. Hyperparameter search spaces used for nested Optuna optimization of SVR and XGBoost. For each outer cross-validation split, Optuna (TPE sampler) minimized inner 3-fold CV RMSE over 25 trials. “Log-uniform” indicates sampling uniformly in log-space. Table S5. Paired statistical comparison of SVR and XGBoost across matched seed/fold evaluations. The table reports paired mean differences, bootstrap 95% confidence intervals, and Wilcoxon signed-rank test p-values for RMSE, MAE, *R* ^2^ and conformal mean interval width across validation regimes. Mean differences are calculated as SVR– XGBoost. Pooled comparisons aggregate matched evaluations across validation regimes and are interpreted as benchmark-level descriptive evidence. Table S6. Full run level results (first 25 rows). Excerpt of the complete run-level output table reporting SVR and XGBoost performance for polymer *T*_g_ prediction across all validation regimes (stratified, scaffold, and fingerprint-cluster splits), random seeds, outer folds, and training fractions. Each row corresponds to a single outer-test evaluation and includes point-performance metrics (RMSE, MAE, *R* ^2^) together with conformal prediction settings (α, nominal coverage) and uncertainty diagnostics (empirical coverage, mean interval width, q_α_). The complete Table S6 is provided as a machine-readable CSV file in the Supporting Information package. Table S7. Column dictionary for Table S6 (full results). Definitions, data types, and units for all fields reported in Table S6, including identifiers for validation regime, model, seed/fold, and training fraction, as well as performance metrics and conformal prediction outputs. This dictionary is provided to facilitate reproducibility and to enable reconstruction of the aggregated statistics reported in the main text and figures. Table S8. Relationships among applicability-domain (AD) diagnostics and prediction error. Table S8A reports pairwise Spearman correlations among AD diagnostics. Table S8B reports Spearman correlations between each AD diagnostic and absolute prediction error for SVR and XGBoost. Values were calculated over pooled repeated-CV outer-test predictions and interpreted descriptively because pooled predictions are not fully independent. Table S9. Threshold-based applicability-domain (AD) diagnostics. Inside- and outside-AD groups were defined using structural similarity, neighborhood-density, descriptor-space, PCA-space, and retrospective target-space support diagnostics. Composite structural AD denotes samples violating at least one structural or descriptor-space AD threshold and excludes target-space diagnostics. Target-space nearest-neighbor distance and outside-training-*T*_g_-range indicators are retrospective only and are not intended for prospective candidate screening. Table S10. Subgroup-level empirical coverage and interval width. Coverage was calculated from per-sample split-conformal intervals at α = 0.10, corresponding to 90% nominal coverage. Each interval corresponds to an outer-test prediction pooled across repeated seed/fold evaluations. Rows report subgroup size, covered intervals, empirical coverage, descriptive 95% binomial confidence intervals, coverage gap relative to nominal coverage, and mean interval width. Because repeated cross-validation produces nonindependent pooled predictions, confidence intervals are provided as descriptive summaries. Table S11. Ranking and top-k selection performance. Ranking fidelity was evaluated using Spearman rank correlation and concordance index. Top-k selection metrics were calculated for identifying high-*T*_g_ candidates using a target threshold of *T*_g_ ≥ 350 K and top-10% selection within each seed/fold unit. Precision@10%, Recall@10%, enrichment factor at 10% selection (EF@10%), and false-positive rate at 10% selection (FPR@10%) are reported. Values for Spearman and C-index are shown as mean ± standard deviation across seed/fold units. The overall rows aggregate all validation regimes and are interpreted as descriptive benchmark-level summaries. Table S12. Interval aware triage analysis under *T*_g_-targeted screening windows. Threshold-based decision utility was evaluated across *T*_g_ targets of 300, 350, and 400 K using pooled outer-test predictions (*N* = 6150). Point-prediction selection used $ŷ\geq T_{g,target}$ , whereas conservative lower-bound selection required $ŷ-q_{\alpha }\geq T_{g,target}$ . In uncertainty-flagged triage, candidates whose conformal intervals overlapped the target threshold were marked as inconclusive; confident positive selections correspond to the lower-bound rule. Precision, recall, and false-positive rate were computed for confident selected candidates. NA indicates undefined precision because no candidates were selected. Table S13. Comparison of XGBoost SHAP and SVR permutation-importance rankings. XGBoost importance is reported as mean absolute SHAP value, whereas SVR permutation importance is reported as the increase in RMSE after descriptor permutation. The two measures are model-specific and are not directly comparable in magnitude. Rankings are interpreted descriptively because correlated descriptors may redistribute importance among related variables (PDF)

## Notes

All scripts required to reproduce the main text and SI results are available on GitHub at https://github.com/muratakdogan06/tg-uq-generalization-benchmark. The repository contains (i) the complete cross-validation benchmarking pipeline for SVR and XGBoost; (ii) RDKit 2D descriptor generation plus five polymer-proxy features; (iii) validation-split generation for stratified, Murcko-scaffold, and Morgan-fingerprint cluster regimes; (iv) split conformal prediction at nominal miscoverage levels α ∈ {0.05, 0.10, 0.20}; (v) maximum test-to-train Morgan–Tanimoto similarity diagnostics (*S*_max_); (vi) XGBoost SHAP importance; and (vii) SI postprocessing scripts under supporting_information/scripts/for paired model comparisons, split characterization, expanded applicability-domain metrics, subgroup conformal coverage, SVR permutation importance, and ranking plus interval-aware triage analyses. For archival reproducibility, cite GitHub release **v1.1.0** corresponding to the submitted manuscript version.
