# Supplementary material for: Trust Beyond Accuracy: Conformal Uncertainty Quantification Reveals the Generalization Gap in Polymer Glass Transition Temperature Prediction
Source: ACS Omega. 2026 Jul 23;11(30):44926–41. doi: 10.1021/acsomega.6c02607 (PMC13448909; doi:10.1021/acsomega.6c02607)
Supplement: Supplementary file 1 [file ao6c02607_si_001.pdf]

Supporting Information

Trust Beyond Accuracy: Conformal Uncertainty Quantification Reveals the Generalization Gap in Polymer Glass Transition Temperature Prediction

Murat Akdoğan[1, *]

1 Hacettepe University, Chemical Engineering Department, Ankara, Turkey

* Corresponding author. Email: muratakdogan@hacettepe.edu.tr

Number of pages: 28

Number of figures: 7

Number of tables: 13

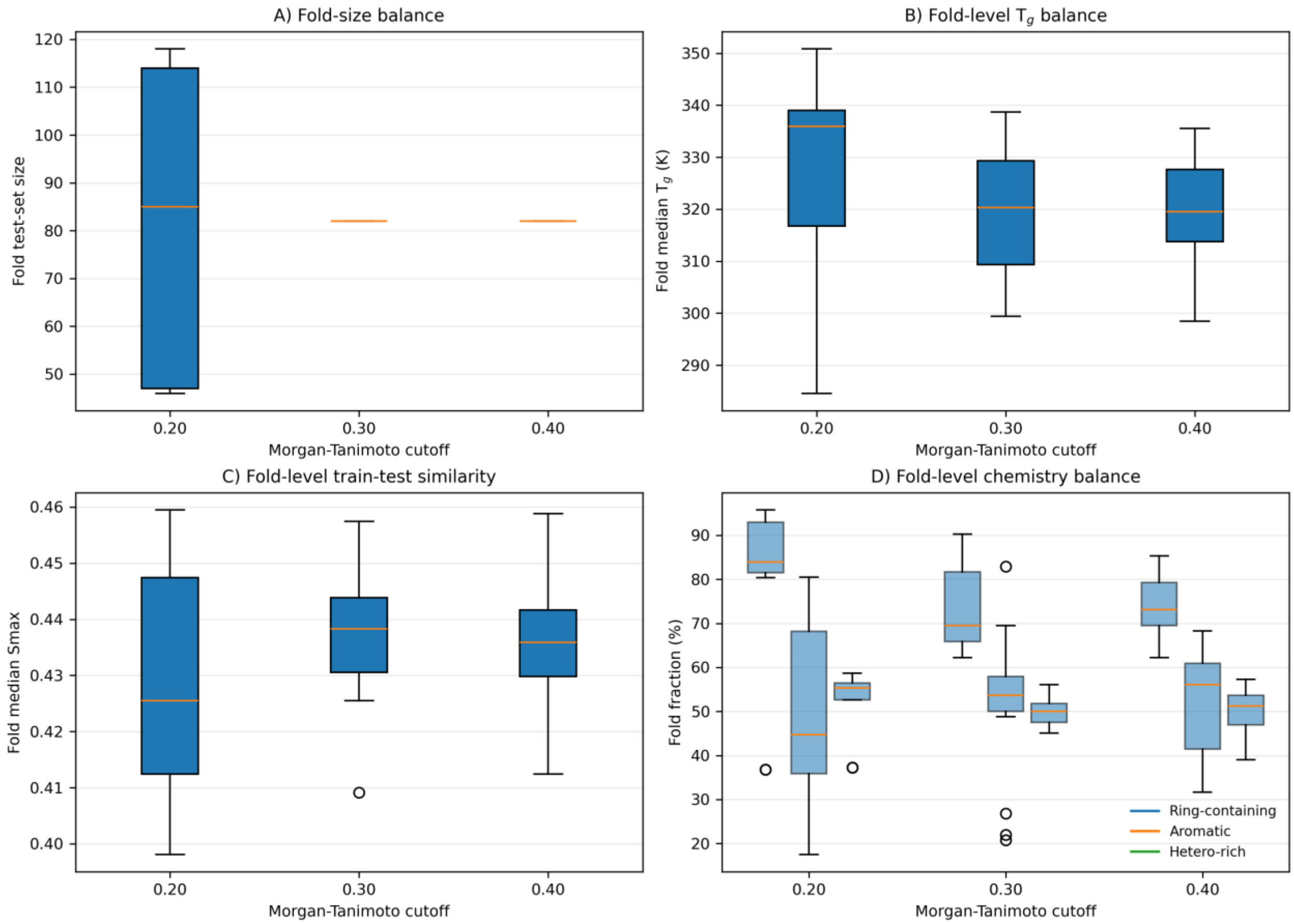

Figure S1. Fold-level T_g and chemistry distribution balance under cluster-based splitting. **(A)** Fold test-set size distribution across Morgan–Tanimoto cutoffs. **(B)** Fold-level median T_g distribution. **(C)** Fold-level median S_{max}, defined as the maximum test-to-train Morgan–Tanimoto similarity. **(D)** Fold-level chemistry-summary distributions, including ring-containing, aromatic, and heteroatom-rich repeat-unit fractions. Boxplots summarize fold-level test-set values across repeated seed/fold partitions.

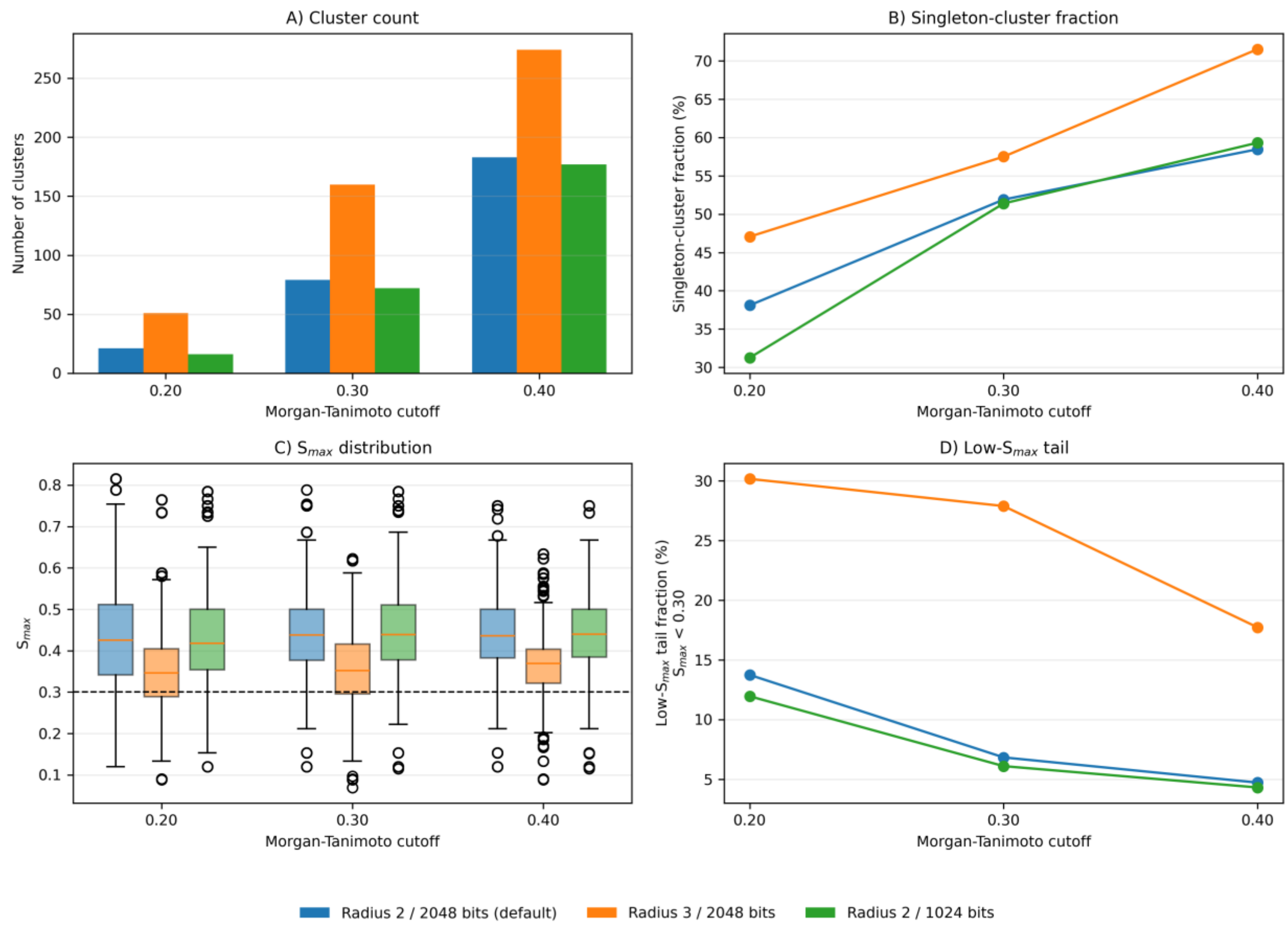

Figure S2. Sensitivity of cluster-split characterization to Morgan fingerprint settings.

Cluster-split diagnostics were recomputed using three Morgan fingerprint configurations: radius 2 / 2048 bits, used as the default setting in the main benchmark; radius 3 / 2048 bits; and radius 2 / 1024 bits. Panels show **(A)** number of clusters, **(B)** singleton-cluster fraction, **(C)** S_{max} distributions, and **(D)** low-S_{max} tail fraction, defined as the fraction of pooled test predictions with $S_{max} < 0.30$. The analysis evaluates the sensitivity of split characterization, not model performance.

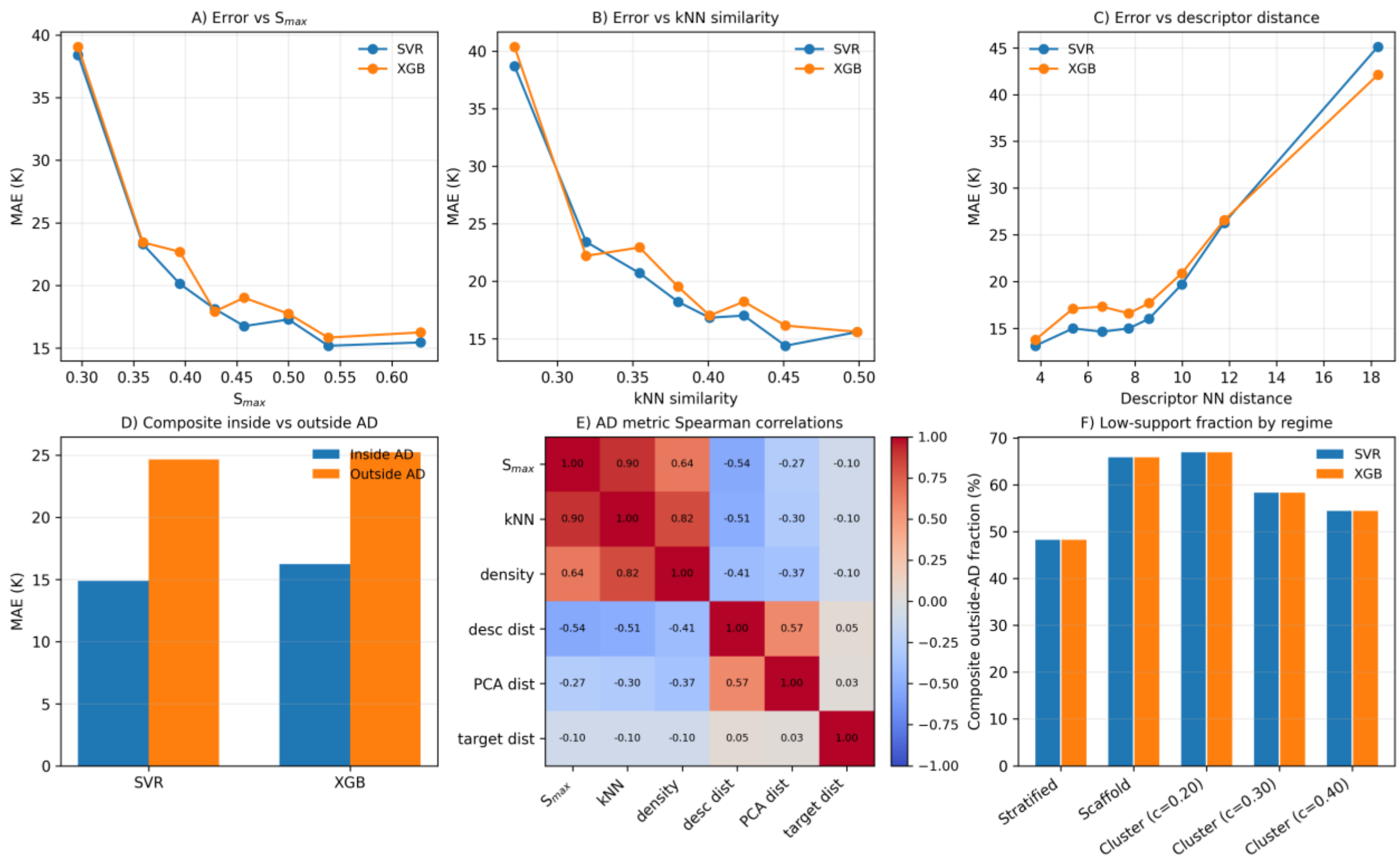

Figure S3. Expanded applicability-domain (AD) diagnostics.

This figure summarizes relationships between prediction error and multiple AD diagnostics, including S_{max}, kNN similarity, descriptor nearest-neighbor distance, composite inside/outside AD status, metric correlations, and outside-AD fraction by validation regime.

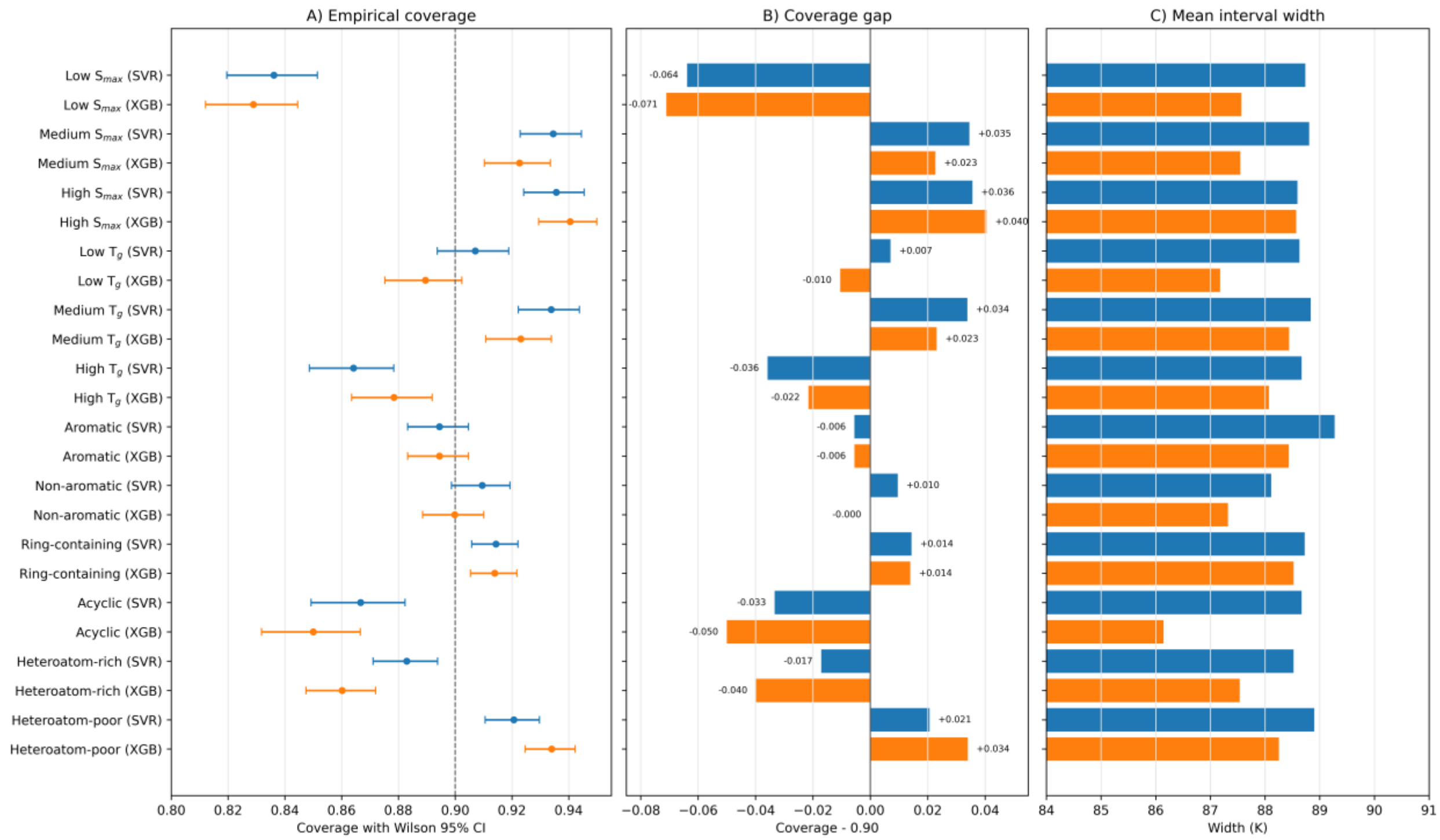

Figure S4. Subgroup-level conformal coverage diagnostics. **(A)** Empirical coverage across S_{max} bins, T_g ranges, and broad repeat-unit chemistry classes at α=0.10. Horizontal error bars indicate descriptive Wilson 95% confidence intervals, and the dashed vertical line indicates nominal coverage. **(B)** Coverage gap relative to 90% nominal coverage; negative values indicate undercoverage and positive values indicate overcoverage. **(C)** Mean conformal interval width across the same subgroups. Blue denotes SVR and orange denotes XGBoost.

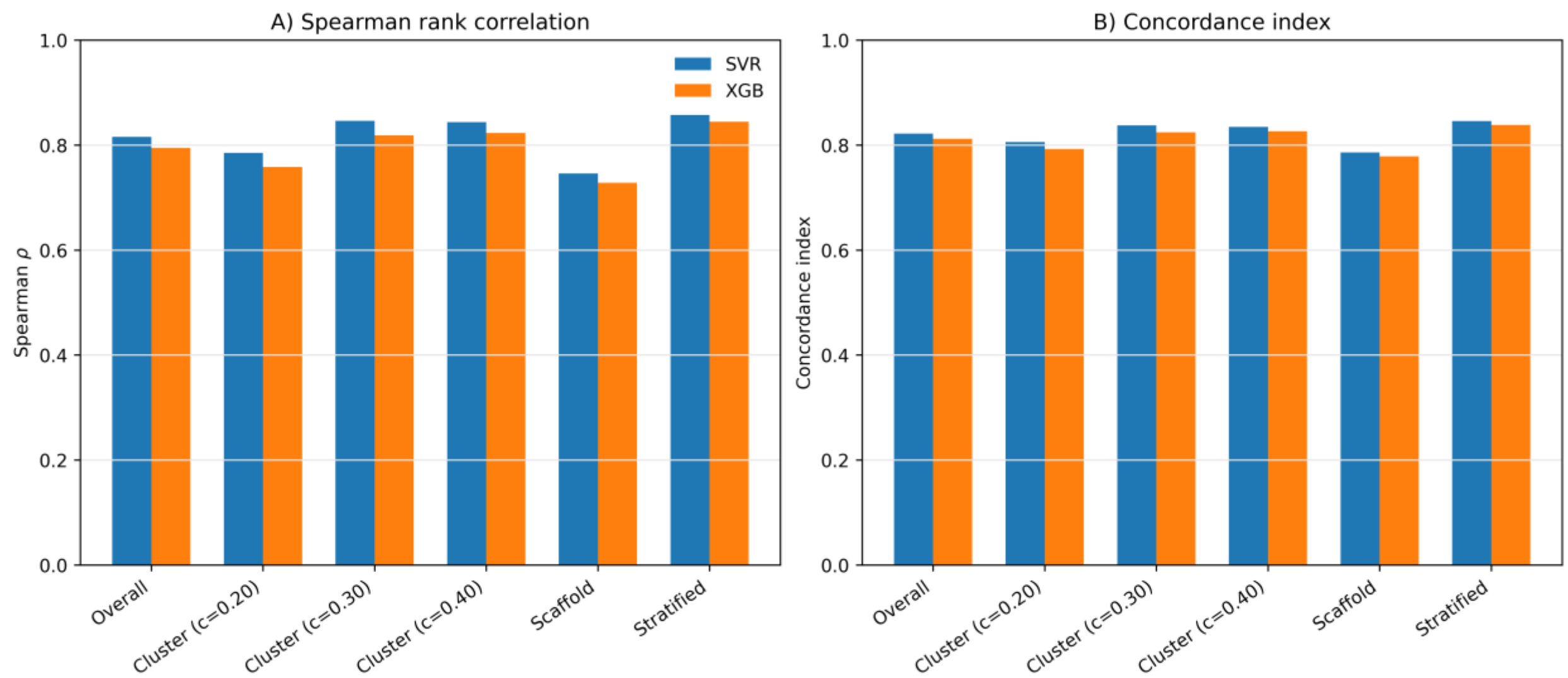

Figure S5. Ranking fidelity across validation regimes.

This figure visualizes Spearman rank correlation and concordance index for SVR and XGBoost across validation regimes.

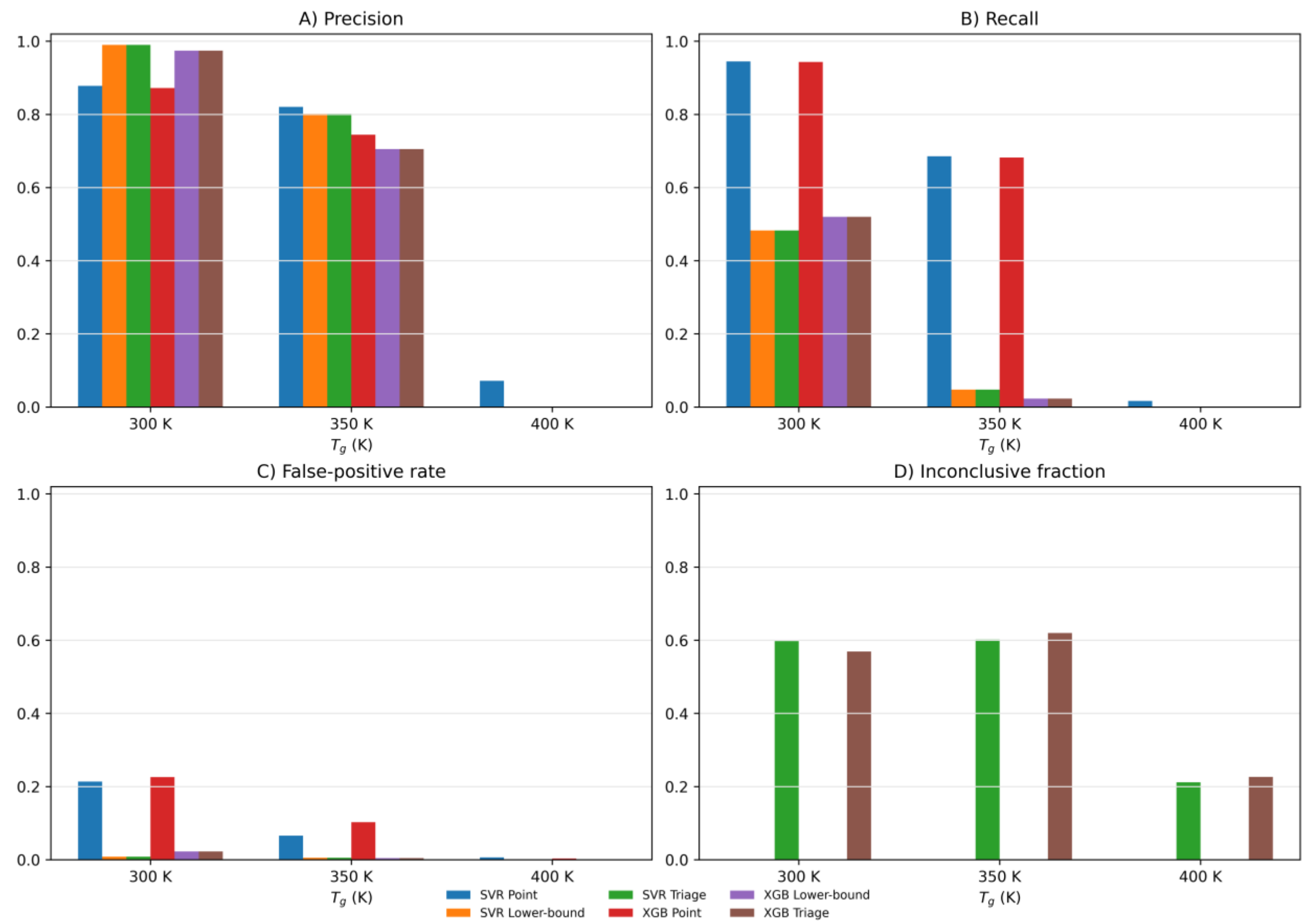

Figure S6. Threshold-based conformal triage for T_g-targeted screening.

This figure visualizes precision, recall, false-positive rate, and inconclusive fraction under point-prediction selection, conservative lower-bound selection, and uncertainty-flagged triage.

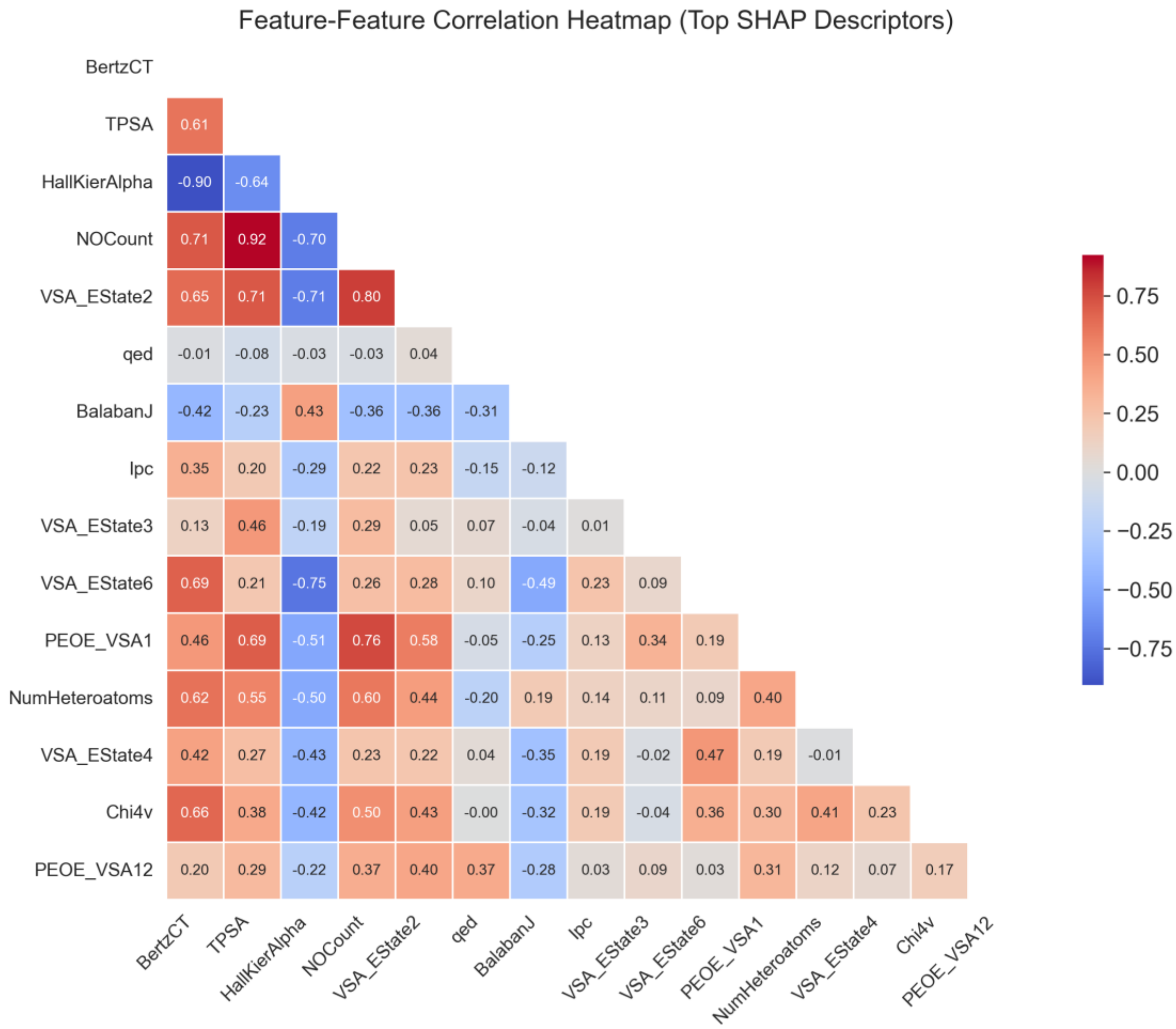

Figure S7. Pearson correlation heatmap for the primary SHAP-ranked descriptors.

Table S1. Custom polymer proxy features included in the predictive representation

Only custom polymer proxy features included in the model are listed. These features were calculated from the processed repeat-unit SMILES using RDKit and custom RDKit-based atom-count routines. Standard RDKit molecular descriptors used in the model are not repeated in this table.

Feature Name	Definition	Unit/Type
proxy_heavy_atoms	Number of heavy atoms, excluding hydrogens, in the repeat-unit molecule.	count; integer
proxy_rings	Number of rings in the repeat-unit molecular graph.	count; integer
proxy_aromatic_frac	Fraction of heavy atoms that are aromatic atoms.	fraction; continuous 0-1
proxy_hetero_frac	Fraction of heavy atoms that are heteroatoms, defined as atoms other than carbon or hydrogen.	fraction; continuous 0-1
proxy_halogen_frac	Fraction of heavy atoms that are halogens: F, Cl, Br, or I.	fraction; continuous 0-1

Table S2. Scaffold characterization for the polymer repeat-unit dataset.

Murcko scaffolds were computed using the same RDKit procedure used for scaffold-based splitting. Empty scaffolds correspond to acyclic repeat units and are intentionally reported because they form a single scaffold group in the scaffold-split diagnostic. Percentages are calculated relative to the full dataset unless otherwise stated in the note column. Generic Murcko scaffold abstraction is reported only as an additional diagnostic of framework compression and was not used for fold assignment. Representative examples are provided for the largest scaffold groups to illustrate the chemical meaning and limitations of scaffold-based partitioning.

**Section**	**Metric or scaffold**	**Value**	**Fraction /denominator (%)**	**Definition / Note**
Overall scaffold diagnostics	Repeat units analyzed	410	100.0	Rows in the frozen processed benchmark dataset.
Overall scaffold diagnostics	Valid RDKit repeat-unit structures	410	100.0	Repeat-unit SMILES successfully parsed by RDKit.
Overall scaffold diagnostics	Unique Murcko scaffold groups, including empty scaffold	145	-	Scaffold grouping exactly follows the scaffold split code.
Overall scaffold diagnostics	Unique non-empty Murcko scaffold groups	144	-	Excludes acyclic repeat units for which RDKit returns an empty Murcko scaffold.
Overall scaffold diagnostics	Largest non-empty Murcko scaffold group	c1ccccc1; 76 repeat units	18.5	Largest non-empty Murcko scaffold group used by the scaffold split; percentage denominator is the full dataset.
Overall scaffold diagnostics	Unique generic Murcko scaffold groups	85	-	Scaffolds after RDKit MakeScaffoldGeneric atom/bond abstraction; empty scaffold retained as one group. Diagnostic only; not used for fold assignment.
Overall scaffold diagnostics	Murcko-to-generic scaffold compression	145 Murcko groups -> 85 generic groups	-	Generic abstraction collapses atom/bond identity and indicates how chemically specific the Murcko labels are; diagnostic only.

Overall scaffold diagnostics	Singleton scaffold groups	115	79.3	Scaffold groups represented by one repeat unit; percentage denominator is unique scaffold groups (115/145).
Overall scaffold diagnostics	Repeat units assigned to singleton scaffolds	115	28.0	Dataset-level singleton scaffold fraction; percentage denominator is all repeat units (115/410).
Overall scaffold diagnostics	Empty Murcko scaffold repeat units	108	26.3	Acyclic repeat units without a Murcko ring framework; the scaffold split groups these together.
Overall scaffold diagnostics	Largest non-empty generic scaffold class	C1CCCCC1; 115 repeat units	28.0	Largest framework after generic Murcko abstraction; useful as a generic-framework concentration diagnostic. Diagnostic only; not used for fold assignment.
Overall scaffold diagnostics	Empty scaffold plus largest generic scaffold class	223 repeat units	54.4	Combined fraction of acyclic repeat units and the dominant generic ring framework class.
Overall scaffold diagnostics	Ring-containing repeat units	302	73.7	Repeat units with RDKit ring count > 0.
Scaffold-size distribution	1 (singleton)	115 scaffold groups; 115 repeat units	28.0	Scaffold group size bin; 79.3% of scaffold groups. Dataset percentage denominator is all repeat units.
Scaffold-size distribution	2	13 scaffold groups; 26 repeat units	6.3	Scaffold group size bin; 9.0% of scaffold groups. Dataset percentage denominator is all repeat units.
Scaffold-size distribution	3-5	9 scaffold groups; 33 repeat units	8.0	Scaffold group size bin; 6.2% of scaffold groups. Dataset percentage denominator is all repeat units.
Scaffold-size distribution	6-10	5 scaffold groups; 35 repeat units	8.5	Scaffold group size bin; 3.4% of scaffold groups. Dataset percentage denominator is all repeat units.
Scaffold-size distribution	>10	3 scaffold groups; 201 repeat units	49.0	Scaffold group size bin; 2.1% of scaffold groups. Dataset percentage denominator is all repeat units.
Representative scaffold examples	[empty scaffold]	108 repeat units	26.3	Generic scaffold: [empty]; example repeat unit: C=CC(=O)OCC(CC)CCCC; Tg range: 203.8-754.5 K.
Representative scaffold examples	c1ccccc1	76 repeat units	18.5	Generic scaffold: C1CCCCC1; example repeat unit: C=C(C)C(=O)Nc1ccccc1OCC; Tg range: 196.5-403.8 K.

Representative scaffold examples	C1CCNCC1	17 repeat units	4.1	Generic scaffold: C1CCCCC1; example repeat unit: C=CC(=O)N1CCC(CNC(=O)CNC(=O)OC(C)(C)C)CC1; Tg range: 251.9-393.2 K.
Representative scaffold examples	C1CC1	10 repeat units	2.4	Generic scaffold: C1CC1; example repeat unit: C=CC(=O)NCC1CC1; Tg range: 225.9-340.6 K.
Representative scaffold examples	C1CCNC1	7 repeat units	1.7	Generic scaffold: C1CCCC1; example repeat unit: C=CC(=O)NCC1CC(C)N(C)C1; Tg range: 282.6-308.0 K.
Representative scaffold examples	C1CNCCN1	6 repeat units	1.5	Generic scaffold: C1CCCCC1; example repeat unit: C=CC(=O)N1CCN(C)C(CC)C1; Tg range: 288.4-376.8 K.
Representative scaffold examples	O=S1(=O)CCCC1	6 repeat units	1.5	Generic scaffold: CC1(C)CCCC1; example repeat unit: C=C(C)C(=O)OC1CCS(=O)(=O)C1; Tg range: 283.9-396.4 K.
Representative scaffold examples	c1ccsc1	6 repeat units	1.5	Generic scaffold: C1CCCC1; example repeat unit: C=CC(=O)Nc1ccsc1C(=O)O; Tg range: 278.6-332.1 K.
Representative scaffold examples	C1CCCCC1	5 repeat units	1.2	Generic scaffold: C1CCCCC1; example repeat unit: C=CC(=O)NCC1CCC(CC)CC1; Tg range: 260.0-337.2 K.
Representative scaffold examples	C1COCCN1	5 repeat units	1.2	Generic scaffold: C1CCCCC1; example repeat unit: C=CC(=O)N1CCOC(C)C1; Tg range: 275.0-345.7 K.

Table S3. Cluster-split characterization across Morgan-Tanimoto cutoffs.

Cluster splits were generated using Morgan fingerprints with radius 2 and 2048 bits. S_{max} denotes the maximum test-to-train Tanimoto similarity. Fold-level summaries are calculated across repeated seed/fold test partitions. Low-S_{max} tail percentages are calculated over pooled outer-test predictions. Chemistry medians summarize fold-level test-set fractions for ring-containing, aromatic, and heteroatom-rich repeat units; heteroatom-rich repeat units were defined using the dataset median heteroatom fraction.

**Morgan-Tanimoto cutoff**	**Number of Clusters**	**Cluster-size distribution**	**Cluster size median [IQR]**	**Largest cluster size**	**Singleton clusters,n (% clusters)**	**Repeat units in singleton clusters, n (% dataset)**	**Fold test-size median [range]**	**Fold median Tg range (K)**	**Fold chemistry medians: ring /aromatic / hetero-rich (%)**	**Smax median [IQR]**	**Low-Smax tail n (%)**
0.20	21	1: 8; 2: 2; 3-5: 2; 6-10: 2; >10: 7	4.0 [1.0, 11.0]	118	8 (38.1)	8 (2.0)	85 [46, 118]	284.6-350.8	83.9/ 44.7 / 55.3	0.426 [0.341, 0.511]	169 (13.7)
0.30	79	1: 41; 2: 12; 3-5: 10; 6-10: 7; >10: 9	1.0 [1.0, 4.0]	59	41 (51.9)	41 (10.0)	82 [82, 82]	299.4-338.7	69.5 /53.7 /50.0	0.438 [0.377, 0.500]	84 (6.8)
0.40	183	1: 107; 2: 36; 3-5: 23; 6-10: 15; >10: 2	1.0 [1.0, 2.0]	17	107 (58.5)	107 (26.1)	82 [82, 82]	298.5-335.6	73.2 /56.1 /51.2	0.436 [0.383, 0.500]	58 (4.7)

Table S4. Hyperparameter search spaces used for nested Optuna optimization of SVR and XGBoost. For each outer cross-validation split, Optuna (TPE sampler) minimized inner 3-fold CV RMSE over 25 trials. “Log-uniform” indicates sampling uniformly in log-space.

	**SVR**	
**Hyperparameter**	**Search space**	**Sampling/ Type**
Kernel	RBF (fixed)	Fixed
C	$10^{-1} – 10^{3}$	Log-uniform
ε	$10^{-3} – 10^{1}$	Log-uniform
γ	$10^{-5} – 10^{0}$	Log-uniform
	**XGBoost**	
**Hyperparameter**	**Search space**	**Sampling/ Type**
max_depth	3 – 10	Integer
learning_rate	0.01 – 0.20	Log-uniform
subsample	0.50 – 1.00	Uniform
colsample_bytree	0.50 – 1.00	Uniform
min_child_weight	10^{-2} – 10	Log-uniform
reg_alpha	10^{-6} – 1	Log-uniform
reg_lambda	10^{-6} – 10	Log-uniform
gamma	10^{-6} – 1	Log-uniform
n_estimators	200 – 1200	Integer

Table S5. Paired statistical comparison of SVR and XGBoost across matched seed/fold evaluations.

The table reports paired mean differences, bootstrap 95% confidence intervals, and Wilcoxon signed-rank test p-values for RMSE, MAE, R^2 and conformal mean interval width across validation regimes. Mean differences are calculated as SVR– XGBoost. Pooled comparisons aggregate matched evaluations across validation regimes and are interpreted as benchmark-level descriptive evidence.

Comparison set	Metric	SVR mean	XGBoost mean	Mean difference (SVR–XGBoost)	Bootstrap 95% CI	Wilcoxon p
Pooled	RMSE (K)	33.44	36.68	-3.24	[-5.11, -1.58]	<0.001
Pooled	MAE (K)	20.68	21.58	-0.90	[-1.54, -0.23]	0.003
Pooled	R^2	0.59	0.47	0.12	[0.05, 0.20]	<0.001
Pooled	Conformal MIW (K)	88.05	88.62	-0.57	[-4.18, 2.92]	0.987
Cluster (c=0.20)	RMSE (K)	36.08	40.44	-4.36	[-8.18, -1.33]	0.010
Cluster (c=0.20)	MAE (K)	21.65	23.45	-1.80	[-2.67, -1.03]	<0.001
Cluster (c=0.20)	R^2	0.54	0.38	0.15	[0.04, 0.31]	0.008
Cluster (c=0.20)	Conformal MIW (K)	84.33	92.23	-7.90	[-17.09, 0.62]	0.188
Cluster (c=0.30)	RMSE (K)	32.94	38.05	-5.11	[-10.15, -1.09]	0.030

Cluster (c=0.30)	MAE (K)	19.89	21.66	-1.76	[-2.89, -0.69]	0.007
Cluster (c=0.30)	R^2	0.62	0.47	0.15	[0.02, 0.32]	0.041
Cluster (c=0.30)	Conformal MIW (K)	88.55	86.48	2.07	[-3.21, 7.02]	0.489
Cluster (c=0.40)	RMSE (K)	33.06	34.66	-1.61	[-4.63, 0.88]	0.489
Cluster (c=0.40)	MAE (K)	20.44	21.02	-0.58	[-1.93, 0.62]	0.847
Cluster (c=0.40)	R^2	0.62	0.57	0.05	[-0.01, 0.14]	0.454
Cluster (c=0.40)	Conformal MIW (K)	87.28	84.37	2.91	[-4.53, 10.55]	0.804
Scaffold	RMSE (K)	32.35	34.90	-2.55	[-8.50, 1.46]	0.762
Scaffold	MAE (K)	21.98	21.21	0.77	[-1.32, 2.91]	0.454
Scaffold	R^2	0.52	0.37	0.16	[-0.03, 0.46]	0.599
Scaffold	Conformal MIW (K)	90.82	89.07	1.75	[-5.03, 8.66]	0.762
Stratified	RMSE (K)	32.77	35.37	-2.59	[-5.96, -0.10]	0.064
Stratified	MAE (K)	19.44	20.57	-1.13	[-2.31, 0.11]	0.121
Stratified	R^2	0.64	0.56	0.07	[0.00, 0.18]	0.083
Stratified	Conformal MIW (K)	89.27	90.96	-1.69	[-10.48, 6.82]	0.720

Table S6. Full run level results (first 25 rows). Excerpt of the complete run-level output table reporting SVR and XGBoost performance for polymer T_g prediction across all validation regimes (stratified, scaffold, and fingerprint-cluster splits), random seeds, outer folds, and training fractions. Each row corresponds to a single outer-test evaluation and includes point-performance metrics (RMSE, MAE, R^2) together with conformal prediction settings (α, nominal coverage) and uncertainty diagnostics (empirical coverage, mean interval width, q_α). The full Table S6 is provided as a machine-readable CSV file in the Supporting Information package.

regime	base_regime	cutoff	model	seed	fold	frac	RMSE	MAE	R2	alpha	nominal_coverage	cov	width	qhat	optuna_best_rmse_innercv
**stratified**	stratified		xgb	42	0	0.2	34.1536	27.0502	0.5563	0.05	0.95	0.8049	92.5385	46.2693	39.1925
**stratified**	stratified		xgb	42	0	0.2	34.1536	27.0502	0.5563	0.1	0.9	0.8049	92.5385	46.2693	39.1925
**stratified**	stratified		xgb	42	0	0.2	34.1536	27.0502	0.5563	0.2	0.8	0.6341	57.2122	28.6061	39.1925
**stratified**	stratified		svr	42	0	0.2	34.6922	26.8616	0.5422	0.05	0.95	0.8780	103.1804	51.5902	40.7954
**stratified**	stratified		svr	42	0	0.2	34.6922	26.8616	0.5422	0.1	0.9	0.8780	103.1804	51.5902	40.7954
**stratified**	stratified		svr	42	0	0.2	34.6922	26.8616	0.5422	0.2	0.8	0.7683	75.6497	37.8249	40.7954
**stratified**	stratified		xgb	42	0	0.4	30.5394	22.9228	0.6452	0.05	0.95	0.9756	158.1693	79.0847	25.1370
**stratified**	stratified		xgb	42	0	0.4	30.5394	22.9228	0.6452	0.1	0.9	0.9634	131.0678	65.5339	25.1370
**stratified**	stratified		xgb	42	0	0.4	30.5394	22.9228	0.6452	0.2	0.8	0.8415	77.8452	38.9226	25.1370
**stratified**	stratified		svr	42	0	0.4	34.3494	24.4372	0.5512	0.05	0.95	0.9390	151.2323	75.6161	22.1565
**stratified**	stratified		svr	42	0	0.4	34.3494	24.4372	0.5512	0.1	0.9	0.9268	131.0464	65.5232	22.1565
**stratified**	stratified		svr	42	0	0.4	34.3494	24.4372	0.5512	0.2	0.8	0.7195	59.7859	29.8929	22.1565
**stratified**	stratified		xgb	42	0	0.6	57.5488	29.0660	-0.2598	0.05	0.95	1.0000	892.0186	446.0093	40.2718
**stratified**	stratified		xgb	42	0	0.6	57.5488	29.0660	-0.2598	0.1	0.9	0.9146	130.3812	65.1906	40.2718
**stratified**	stratified		xgb	42	0	0.6	57.5488	29.0660	-0.2598	0.2	0.8	0.7561	66.4979	33.2490	40.2718
**stratified**	stratified		svr	42	0	0.6	36.8816	24.6577	0.4826	0.05	0.95	0.9024	120.9491	60.4746	36.5156
**stratified**	stratified		svr	42	0	0.6	36.8816	24.6577	0.4826	0.1	0.9	0.8780	101.7611	50.8806	36.5156
**stratified**	stratified		svr	42	0	0.6	36.8816	24.6577	0.4826	0.2	0.8	0.7927	74.2072	37.1036	36.5156
**stratified**	stratified		xgb	42	0	0.8	31.5468	24.3399	0.6214	0.05	0.95	0.9024	98.4503	49.2252	38.3010
**stratified**	stratified		xgb	42	0	0.8	31.5468	24.3399	0.6214	0.1	0.9	0.8537	84.4766	42.2383	38.3010
**stratified**	stratified		xgb	42	0	0.8	31.5468	24.3399	0.6214	0.2	0.8	0.8171	68.2679	34.1340	38.3010
**stratified**	stratified		svr	42	0	0.8	32.5496	23.2832	0.5970	0.05	0.95	0.8780	103.6221	51.8111	36.2060
**stratified**	stratified		svr	42	0	0.8	32.5496	23.2832	0.5970	0.1	0.9	0.8537	86.5010	43.2505	36.2060
**stratified**	stratified		svr	42	0	0.8	32.5496	23.2832	0.5970	0.2	0.8	0.8415	79.3867	39.6933	36.2060
**stratified**	stratified		xgb	42	0	1	29.8088	22.5986	0.6620	0.05	0.95	0.8537	86.5232	43.2616	38.9739

Table S7. Column dictionary for Table S6 (full results). Definitions, data types, and units for all fields reported in Table S6, including identifiers for validation regime, model, seed/fold, and training fraction, as well as performance metrics and conformal prediction outputs. This dictionary is provided to facilitate reproducibility and to enable reconstruction of the aggregated statistics reported in the main text and figures.

Column	Type	Units	Level	Description
regime	object		run level	Composite evaluation regime label used in file outputs (may include cutoff).
base_regime	object		run level	Regime family without cutoff detail: stratified, scaffold, or cluster.
cutoff	float64	Tanimoto distance (unitless)	run level	Cluster splitting Tanimoto distance cutoff (only for cluster regimes; blank/NaN otherwise).
model	object		run level	Model class evaluated (svr or xgb).
seed	int64	-	run level	Random seed used to generate the outer split and subsampling.
fold	int64	-	run level	Outer cross-validation fold index (1-5).
frac	float64	fraction	run level	Training fraction used for learning-curve subsampling (0.2–1.0).
RMSE	float64	K	run level	Root mean squared error on the outer test fold.
MAE	float64	K	run level	Mean absolute error on the outer test fold.
R^2	float64	unitless	run level	Coefficient of determination on the outer test fold.
optuna_best_rmse_innercv	float64	K	run level	Best inner-CV RMSE achieved by Optuna during hyperparameter tuning for the given outer fold.

alpha	float64	fraction	run level	Conformal miscoverage level used for split conformal prediction (e.g., 0.05, 0.10, 0.20).
nominal_coverage	float64	fraction	run level	Nominal target coverage corresponding to alpha (1 − alpha).
cov	float64	fraction	run level	Empirical coverage on the outer test fold (fraction of y within the conformal interval).
width	float64	K	run level	Mean conformal prediction-interval width on the outer test fold.
qhat	float64	K	run level	Conformal half-width (quantile of calibration residuals) used to form symmetric intervals.

Table S8. Relationships among applicability-domain (AD) diagnostics and prediction error. Part A reports pairwise Spearman correlations among AD diagnostics. Part B reports Spearman correlations between each AD diagnostic and absolute prediction error for SVR and XGBoost. Values were calculated over pooled repeated-CV outer-test predictions and interpreted descriptively because pooled predictions are not fully independent.

(A) Pairwise Spearman correlations among AD diagnostics

**AD metric**	**S_{max}**	**kNN similarity**	**Tanimoto density**	**Descriptor NN distance**	**PCA distance**	**Target-space NN distance**
S_{max}	1.00	0.90	0.64	-0.54	-0.27	-0.10
kNN similarity	0.90	1.00	0.82	-0.51	-0.30	-0.10
Tanimoto density	0.64	0.82	1.00	-0.41	-0.37	-0.10
Descriptor NN distance	-0.54	-0.51	-0.41	1.00	0.57	0.05
PCA distance	-0.27	-0.30	-0.37	0.57	1.00	0.03
Target-space NN distance	-0.10	-0.10	-0.10	0.05	0.03	1.00

(B) Spearman correlations between AD diagnostics and absolute prediction error.

**AD diagnostic**	**Diagnostic type**	**Expected risk direction**	**SVR Spearman ρ with absolute error**	**XGBoost Spearman ρ with absolute error**
S_{max}	Structural similarity	Lower S_{max} -> higher error	-0.209	-0.187

kNN similarity	Local structural support	Lower kNN similarity -> higher error	-0.221	-0.196
Tanimoto density	Neighborhood density	Lower density -> higher error	-0.229	-0.193
Descriptor NN distance	Descriptor-space support	Higher distance -> higher error	0.327	0.243
PCA distance	Descriptor-space support	Higher distance -> higher error	0.237	0.206
Target-space NN distance	Retrospective target support	Higher distance -> higher error	0.076	0.076
Outside training T_g range	Retrospective target support	Outside range -> higher error	0.125	0.119

Table S9. Threshold-based applicability-domain (AD) diagnostics.

Inside- and outside-AD groups were defined using structural similarity, neighborhood-density, descriptor-space, PCA-space, and retrospective target-space support diagnostics. Composite structural AD denotes samples violating at least one structural or descriptor-space AD threshold and excludes target-space diagnostics. Target-space nearest-neighbor distance and outside-training-T_g-range indicators are retrospective only and are not intended for prospective candidate screening.

**AD diagnostic**	**Diagnostic type**	**Inside-AD rule**	**Outside, n (%)**	**SVR MAE inside / outside**	**XGBoost MAE inside / outside**	**SVR coverage inside / outside**	**XGBoost coverage inside / outside**
S_{max}	Structural similarity	$S_{max} \geq$ 0.300	444 (7.2%)	18.4 / 48.5	19.2 / 51.4	0.917 / 0.707	0.910 / 0.725
kNN similarity	Local structural support	kNN similarity ≥ 0.338	1538 (25.0%)	17.1 / 31.1	18.2 / 31.3	0.935 / 0.803	0.924 / 0.815
Tanimoto density	Neighborhood density	Density ≥ 0.017	1535 (25.0%)	17.2 / 30.9	18.3 / 31.2	0.935 / 0.802	0.924 / 0.817
Descriptor NN distance	Descriptor-space support	Distance ≤ 10.717	1538 (25.0%)	15.6 / 35.7	17.2 / 34.3	0.954 / 0.745	0.939 / 0.770
PCA distance	Descriptor-space support	Distance ≤ 3.216	1538 (25.0%)	16.3 / 33.5	17.4 / 33.7	0.948 / 0.764	0.937 / 0.776
Composite structural AD	Composite structural support	No structural AD threshold violated	3614 (58.8%)	14.9 / 24.6	16.2 / 25.2	0.961 / 0.860	0.957 / 0.855
Target-space NN distance*	Retrospective target support	Distance ≤ 0.446 K	1536 (25.0%)	18.4 / 27.2	19.1 / 28.6	0.920 / 0.848	0.913 / 0.850
Outside training T_g range*	Retrospective target support	T_g within training-fold target range	36 (0.6%)	19.2 / 264.5	19.8 / 304.7	0.906 / 0.139	0.902 / 0.111

Table S10. Subgroup-level empirical coverage and interval width.

Coverage was calculated from per-sample split-conformal intervals at α=0.10, corresponding to 90% nominal coverage. Each interval corresponds to an outer-test prediction pooled across repeated seed/fold evaluations. Rows report subgroup size, covered intervals, empirical coverage, descriptive 95% binomial confidence intervals, coverage gap relative to nominal coverage, and mean interval width. Because repeated cross-validation produces non-independent pooled predictions, confidence intervals are provided as descriptive summaries.

Model	Subgroup family	Subgroup	Intervals	Covered	Empirical coverage	95% binomial CI	Coverage gap	MIW (K)
SVR	S_{max} bins	low	2069	1730	0.836	[0.820, 0.851]	-0.064	88.74
SVR	S_{max} bins	medium	2031	1898	0.935	[0.923, 0.944]	+0.035	88.81
SVR	S_{max} bins	high	2050	1918	0.936	[0.924, 0.945]	+0.036	88.59
SVR	T_g range	low	2055	1864	0.907	[0.894, 0.919]	+0.007	88.63
SVR	T_g range	medium	2055	1919	0.934	[0.922, 0.944]	+0.034	88.84
SVR	T_g range	high	2040	1763	0.864	[0.849, 0.878]	-0.036	88.67
SVR	Chemistry: aromaticity	aromatic	3165	2831	0.894	[0.883, 0.905]	-0.006	89.27
SVR	Chemistry: aromaticity	non-aromatic	2985	2715	0.910	[0.899, 0.919]	+0.010	88.11
SVR	Chemistry: rings	ring-containing	4530	4142	0.914	[0.906, 0.922]	+0.014	88.73

SVR	Chemistry: rings	acyclic	1620	1404	0.867	[0.849, 0.882]	-0.033	88.67
SVR	Chemistry: heteroatom content	heteroatom-rich	3075	2715	0.883	[0.871, 0.894]	-0.017	88.52
SVR	Chemistry: heteroatom content	heteroatom-poor	3075	2831	0.921	[0.911, 0.930]	+0.021	88.90
XGBoost	S_{max} bins	low	2069	1715	0.829	[0.812, 0.845]	-0.071	87.57
XGBoost	S_{max} bins	medium	2031	1874	0.923	[0.910, 0.934]	+0.023	87.55
XGBoost	S_{max} bins	high	2050	1928	0.940	[0.929, 0.950]	+0.040	88.57
XGBoost	T_g range	low	2055	1828	0.890	[0.875, 0.902]	-0.010	87.18
XGBoost	T_g range	medium	2055	1897	0.923	[0.911, 0.934]	+0.023	88.44
XGBoost	T_g range	high	2040	1792	0.878	[0.864, 0.892]	-0.022	88.07
XGBoost	Chemistry: aromaticity	aromatic	3165	2831	0.894	[0.883, 0.905]	-0.006	88.44
XGBoost	Chemistry: aromaticity	non-aromatic	2985	2686	0.900	[0.889, 0.910]	0.000	87.32
XGBoost	Chemistry: rings	ring-containing	4530	4140	0.914	[0.905, 0.922]	+0.014	88.52
XGBoost	Chemistry: rings	acyclic	1620	1377	0.850	[0.832, 0.867]	-0.050	86.14
XGBoost	Chemistry: heteroatom content	heteroatom-rich	3075	2645	0.860	[0.847, 0.872]	-0.040	87.54
XGBoost	Chemistry: heteroatom content	heteroatom-poor	3075	2872	0.934	[0.925, 0.942]	+0.034	88.25

Table S11. Ranking and top-k selection performance.

Ranking fidelity was evaluated using Spearman rank correlation and concordance index. Top-k selection metrics were calculated for identifying high-Tg candidates using a target threshold of $T_g \geq 350$ K and top-10% selection within each seed/fold unit. Precision@10%, Recall@10%, enrichment factor at 10% selection (EF@10%), and false-positive rate at 10% selection (FPR@10%) are reported. Values for Spearman and C-index are shown as mean ± standard deviation across seed/fold units. The overall rows aggregate all validation regimes and are interpreted as descriptive benchmark-level summaries.

**Comparison set**	**Model**	**Seed/fold units**	**Spearman mean ± SD**	**C-index mean± SD**	**Precision@10%**	**Recall@10%**	**EF@10%**	**FPR@10%**
Pooled	SVR	75	0.816 ± 0.062	0.822 ± 0.030	0.785	0.291	2.687	0.031
Pooled	XGBoost	75	0.795 ± 0.077	0.812 ± 0.037	0.782	0.290	2.676	0.032
Cluster (c=0.20)	SVR	15	0.785 ± 0.058	0.806 ± 0.026	0.679	0.258	2.443	0.045
Cluster (c=0.20)	XGBoost	15	0.758 ± 0.080	0.793 ± 0.036	0.683	0.261	2.468	0.045
Cluster (c=0.30)	SVR	15	0.846 ± 0.030	0.837 ± 0.013	0.852	0.321	2.925	0.022
Cluster (c=0.30)	XGBoost	15	0.818 ± 0.034	0.824 ± 0.016	0.844	0.324	2.949	0.024
Cluster (c=0.40)	SVR	15	0.843 ± 0.044	0.834 ± 0.024	0.807	0.296	2.700	0.030
Cluster (c=0.40)	XGBoost	15	0.823 ± 0.065	0.826 ± 0.034	0.844	0.312	2.843	0.025
Scaffold	SVR	15	0.746 ± 0.058	0.786 ± 0.023	0.705	0.260	2.478	0.041
Scaffold	XGBoost	15	0.728 ± 0.091	0.779 ± 0.039	0.672	0.239	2.277	0.043
Stratified	SVR	15	0.857 ± 0.028	0.846 ± 0.015	0.881	0.317	2.891	0.019
Stratified	XGBoost	15	0.845 ± 0.037	0.838 ± 0.016	0.867	0.312	2.843	0.021

Table S12. Interval aware triage analysis under T_g-targeted screening windows.

Threshold-based decision utility was evaluated across T_g targets of 300, 350, and 400 K using pooled outer-test predictions (N = 6150). Point-prediction selection used $\hat{y} \geq T_{g,\text{target}}$, whereas conservative lower-bound selection required $\hat{y} - q_\alpha \geq T_{g,\text{target}}$. In uncertainty-flagged triage, candidates whose conformal intervals overlapped the target threshold were marked as inconclusive; confident positive selections correspond to the lower-bound rule. Precision, recall, and false-positive rate were computed for confident selected candidates. NA indicates undefined precision because no candidates were selected.

**Model**	**Target T_g (K)**	**True positives available**	**Point: selected / precision / recall / FPR**	**Lower-bound: selected / precision / recall / FPR**	**Inconclusive n (%)**
SVR	300	3810	4100/ 0.878 / 0.945 / 0.214	1857 / 0.990 / 0.482 / 0.008	3676 (59.8%)
SVR	350	1875	1566 /0.821 / 0.685 /0.066	111 / 0.802 / 0.047 / 0.005	3702 (60.2%)
SVR	400	180	42 / 0.071 / 0.017 / 0.007	0 / NA / 0.000 / 0.000	1301 (21.2%)
XGBoost	300	3810	4121 /0.872 / 0.943 / 0.226	2034 / 0.974 / 0.520 / 0.023	3499 (56.9%)
XGBoost	350	1875	1718 / 0.744 / 0.682 / 0.103	61 / 0.705 / 0.023 / 0.004	3812 (62.0%)
XGBoost	400	180	21 / 0.000 / 0.000 /0.004	7 / 0.000 / 0.000 / 0.001	1392 (22.6%)

Table S13. Comparison of XGBoost SHAP and SVR permutation-importance rankings. XGBoost importance is reported as mean absolute SHAP value, whereas SVR permutation importance is reported as the increase in RMSE after descriptor permutation. The two measures are model-specific and are not directly comparable in magnitude. Rankings are interpreted descriptively because correlated descriptors may redistribute importance among related variables.

Feature	XGBoost SHAP rank	XGBoost mean |SHAP|	SVR permutation rank	SVR RMSE increase	SVR RMSE increase SD
BCUT2D_MRLOW	34	0.3710	1	1.3807	0.0844
BertzCT	1	8.7211	49	0.1595	0.0477
MinAbsEStateIndex	71	0.1454	2	0.6915	0.0965
TPSA	2	5.6309	7	0.4331	0.0875
HallKierAlpha	3	5.1247	28	0.2008	0.0487
SMR_VSA2	191	0.0000	3	0.5359	0.1422
NOCount	4	4.6071	9	0.3684	0.0752
fr_nitrile	149	0.0000	4	0.5359	0.1422
BCUT2D_LOGPLOW	17	0.8608	5	0.4871	0.0562
VSA_EState2	5	3.7399	19	0.2514	0.0530
BCUT2D_CHGLO	65	0.1623	6	0.4496	0.0606
qed	6	2.4856	51	0.1586	0.0406
BalabanJ	7	2.3235	185	0.0372	0.0174
Ipc	8	1.7778	66	0.1325	0.1299
PEOE_VSA12	15	0.9476	8	0.3718	0.0657
VSA_EState3	9	1.4125	57	0.1518	0.0479
VSA_EState6	10	1.3828	134	0.0895	0.0286
fr_quatN	154	0.0000	10	0.3495	0.1268

NumHDonors	61	0.2126	11	0.3210	0.0660
PEOE_VSA1	11	1.3774	16	0.2787	0.0495
NHOHCount	23	0.6056	12	0.3170	0.0661
NumHeteroatoms	12	1.2009	64	0.1343	0.0447
VSA_EState4	13	1.1036	61	0.1382	0.0561
fr_priamide	161	0.0000	13	0.3055	0.0948
BCUT2D_CHGHI	55	0.2237	14	0.2869	0.0528
Chi4v	14	1.0672	164	0.0678	0.0316
fr_ketone	138	0.0000	15	0.2798	0.0715
PEOE_VSA8	16	0.8681	145	0.0811	0.0358
fr_amide	27	0.5080	17	0.2567	0.0582
NumAmideBonds	19	0.7205	18	0.2567	0.0582
SlogP_VSA2	18	0.8307	102	0.1103	0.0300
BCUT2D_LOGPHI	91	0.0827	20	0.2457	0.0498
PEOE_VSA2	20	0.6461	40	0.1672	0.0353
Chi2v	21	0.6439	159	0.0746	0.0273
proxy_hetero_frac	40	0.3371	21	0.2387	0.0708
MaxAbsEStateIndex	47	0.3019	22	0.2386	0.0484
NumHeterocycles	22	0.6074	122	0.0979	0.0407
MaxEStateIndex	72	0.1388	23	0.2386	0.0484
fr_NH0	24	0.5208	107	0.1077	0.0411
fr_sulfide	153	0.0000	24	0.2205	0.1168
RingCount	25	0.5152	88	0.1225	0.0388
fr_bicyclic	128	0.0081	25	0.2051	0.0534
